# Psychotic relapse prediction via biomarker monitoring: a systematic review

**DOI:** 10.3389/fpsyt.2024.1463974

**Published:** 2024-12-03

**Authors:** Alexandros Smyrnis, Christos Theleritis, Panagiotis Ferentinos, Nikolaos Smyrnis

**Affiliations:** ^1^ Laboratory of Cognitive Neuroscience and Sensorimotor Control, University Mental Health, Neurosciences and Precision Medicine Research Institute “COSTAS STEFANIS”, Athens, Greece; ^2^ 2^nd^Psychiatry Department, National and Kapodistrian University of Athens, Medical School, University General Hospital “ATTIKON”, Athens, Greece

**Keywords:** digital phenotyping, genetic biomarker, cognitive biomarker, neuroimaging biomarker, blood-based biomarker, psychosis

## Abstract

**Background:**

Associating temporal variation of biomarkers with the onset of psychotic relapse could help demystify the pathogenesis of psychosis as a pathological brain state, while allowing for timely intervention, thus ameliorating clinical outcome. In this systematic review, we evaluated the predictive accuracy of a broad spectrum of biomarkers for psychotic relapse. We also underline methodological concerns, focusing on the value of prospective studies for relapse onset estimation.

**Methods:**

Following the PRISMA (Preferred Reporting Items for Systematic Review and Meta-Analysis) guidelines, a list of search strings related to biomarkers and relapse was assimilated and run against the PubMed and Scopus databases, yielding a total of 808 unique records. After exclusion of studies related to the distinction of patients from controls or treatment effects, the 42 remaining studies were divided into 5 groups, based on the type of biomarker used as a predictor: the genetic biomarker subgroup (n = 4, or 9%), the blood-based biomarker subgroup (n = 15, or 36%), the neuroimaging biomarker subgroup (n = 10, or 24%), the cognitive-behavioral biomarker subgroup (n = 5, or 12%) and the wearables biomarker subgroup (n = 8, or 19%).

**Results:**

In the first 4 groups, several factors were found to correlate with the state of relapse, such as the genetic risk profile, Interleukin-6, Vitamin D or panels consisting of multiple markers (blood-based), ventricular volume, grey matter volume in the right hippocampus, various functional connectivity metrics (neuroimaging), working memory and executive function (cognition). In the wearables group, machine learning models were trained based on features such as heart rate, acceleration, and geolocation, which were measured continuously. While the achieved predictive accuracy differed compared to chance, its power was moderate (max reported AUC = 0.77).

**Discussion:**

The first 4 groups revealed risk factors, but cross-sectional designs or sparse sampling in prospective studies did not allow for relapse onset estimations. Studies involving wearables provide more concrete predictions of relapse but utilized markers such as geolocation do not advance pathophysiological understanding. A combination of the two approaches is warranted to fully understand and predict relapse.

## 1 Introduction

The etiopathological underpinnings of psychosis, defined as a pathological brain state (1), as well as psychotic disorders as diagnostic constructs, still elude us after more than 50 years of research (2). Apart from their inherent heterogeneity regarding clinical manifestations, it is challenging to demystify the causality of psychotic disorders due to their seemingly random onset, chronic course and recurrent nature, which leaves a lasting and progressive impact on patient functioning. Crucially, over 80% of individuals with psychotic disorders will experience relapses (3), or transitions to a state of psychosis. The current best approach to prevent them is via continuation of antipsychotic and/or mood stabilizing treatment for years (4), which then exposes patients to a variety of serious medication side effects. It is established in the literature that this leads to issues regarding compliance (5), while treatment non-adherence has been shown to be the single most significant predictor of relapse (6). Furthermore, even among those who follow treatment, there is still a 20-30% chance of symptom recurrence after First-Episode Psychosis (FEP) (7). From a clinical perspective, early identification of psychotic relapse would be of vital importance since the clinician would then be able to stop the vicious cycle of symptom recurrence after treatment discontinuation.

Nevertheless, an overwhelming percentage of studies in the field of psychotic disorders revolve around distinguishing between patients and healthy controls. The most prominent study design includes a cross-sectional comparison of a potentially implicated etiological factor between patients and healthy controls, or between patients with different diagnoses. While this approach has unraveled several risk factors for psychotic disorders, it does not allow for predictions regarding the course of the disease for individual patients, therefore providing limited clinical benefits. Studies involving relapse, on the other hand, could shed light on possible deciders of disease course, but are significantly harder to design and perform. Given the chronicity and random trajectory of the phenomenon, these studies must be prospective, while ideally measurements or monitoring need to be close to continuous, to have available data at, or around the time of relapse, to draw comparisons with data originating from periods of remission. Additionally, the amount of data required to be amassed for a sufficient number of relapse events to be recorded is massive, given their relative sparsity (an epidemiological study (8) measured 751 events in 3980-participant years). Analyzing such a long-term phenomenon entails diligent patient monitoring for years. Another caveat that needs to be accounted for, is that treatment adherence cannot be controlled in such studies. The lack of a scalable way to confirm medication status (9) at relapse introduces confounding factors that could hinder result reliability.

Despite these obstacles, there have been attempts to map the course of psychotic disorders and identify potential risk factors, or predictors for relapse. The vast majority of these efforts involve models with solely clinical variables as predictors and include no biological factors (or biomarkers) [see (10) for a meta-review]. Treatment non-adherence and premorbid functioning have been isolated as the most significant predictors of relapse. However, two distinct issues arise when developing exclusively clinical prediction models. Firstly, little to no new insight is gained regarding pathophysiology, thus no progress can be achieved regarding intervention effectiveness and new medications. Additionally, clinical models provide no information regarding the exact temporal onset of a relapse occurrence, which would allow for early intervention. Clinical variables such as family history, alcohol consumption, or drug abuse are represented as binary variables measured at one instance in time (cross sectional design). Other variables, such as premorbid functioning do not evolve at all in time. Yet the course of psychotic disorders is dynamic in time, characterized by psychotic episodes followed by remission phases. Moreover, given the relatively sudden onset of symptom recurrence, it would be reasonable to assume some biological change happening on short time scales. To capture it, one would have to monitor some biological factors, or biomarkers, at a sufficiently high frequency. The term biomarker is defined as “a characteristic that is objectively measured and evaluated as an indicator of normal biological processes, pathogenic processes, or pharmacologic responses to a therapeutic intervention” (11), and it could refer to anything from the serum concentration of a specific hormone to the time elapsed when a human responds to some stimulus [Reaction Time). Notably, biomarkers evolve on various time scales, ranging from milliseconds to hours or even days, in stark contrast to clinical markers. Heart rate, for example, has been shown to exhibit variability on very short time scales (in the 0.15 - 0.4 Hz frequency range (12)], but also fluctuates diurnally, especially between night and day time (13). To conclude, clinical variables seem unsuitable for predicting the temporal onset of relapse, whereas the same cannot be said for biomarkers (14), whose short-term alteration could correlate with symptom reignition.

It becomes apparent that the utilization of biomarkers in predictive models for psychotic relapse, either exclusively or in conjunction with clinical parameters, could provide the missing piece to solve the conundrum in hand. In this systematic review, we consolidate and present the findings of all studies using genetic, blood-based, neuroimaging, cognitive and behavioral biomarkers as predictors for psychotic relapse. We also cover a distinct category of studies in which data is continuously accrued via wearable devices or smartphones. Data from these studies includes accelerometer or heart rate measurements, which are commonly used biomarkers, but also information regarding geolocation, text messages, duration of phone calls, or screen activity, which we consider as proxies of behavior. The detailed inclusion criteria are reported in the methods section, but to outline the process, we included studies that longitudinally monitored biomarker levels and clinically evaluated patients to identify relapse. Biomarker levels were either measured continuously, or at two or more distinct time points (usually with one corresponding to a period of relative health and one corresponding to relapse). We included cross sectional studies if and only if the entire sample consisted of patients experiencing symptom recurrence, and not first-episode psychosis. The objective of the present review is to delineate the progress that has been accomplished so far regarding relapse prediction via biomarker monitoring, but also to underline potential methodological caveats.

## 2 Methods

### 2.1 Main outcome

The main outcome of this study is psychotic relapse, which is defined clinically, and refers to the occurrence of a noninitial psychotic episode, after a period of symptom remission. We largely base our definition of remission on Andreassen’s criteria (20), where the authors propose a clinical framework for defining remission in SCZ based on score thresholds in the Positive and Negative Symptom Scale (PANNS), for items such as P1 (Delusions), P2 (Conceptual Disorganization), P3 (Hallucinatory behavior), and G9 (Unusual thought content), as well as in the Brief Psychiatric Rating Scale (BRPS), regarding items 8(Grandiosity), 11 (Suspiciousness), 12 (Hallucinatory behavior) and others. Andreassen et al. suggest that these scores must remain at below-threshold levels for 6 months for remission to be defined, but we impose the lower bound of 1 month in the present review. Moreover, we deemed that if patients were discharged from the hospital after a clinical evaluation, it is implied that they entered a period of potential remission, even if the actual scores of the evaluations were not reported. We only excluded studies that treated rehospitalizations as adverse outcomes, with no mention of SCZ diagnosis or psychotic symptomatology as the reason for readmission to the hospital. The relative leniency of these criteria is due to the objective of this study, which is to bring the findings of biomarkers research to the forefront. Given the state of the field, we do not believe it is yet appropriate to formulate standardized guidelines, which would be directly applied in clinical practice.

### 2.2 Study design overview

This systematic review was conducted in alignment with the PRISMA (Preferred Reporting Items for Systematic Reviews and Meta-Analyses) guidelines [ (15, 16), see **Supplementary Materials**]. The selection process of articles that were included broadly consisted of three phases, which started in February of 2024 and were concluded in April of the same year. During the first step of the process, a list of relevant keywords were identified, which were then run against records in the PubMed and Scopus databases. We also hand-checked citations of all retrieved papers, obtaining no new, unique records. Search results were then screened (title and abstract initially, then full papers) based on a set of inclusion and exclusion criteria tailored to the PICOS/PECOS worksheet (15, 16). The final step included a categorization of studies into subgroups according to the nature of examined biomarkers, namely genetic, blood-based, neuroimaging, cognitive, behavioral and related to wearable devices and smartphones. Studies were then meticulously analyzed, and relevant information was distilled in the form of tables.

### 2.3 Selection and analysis procedure

To begin with, PubMed and Scopus databases were searched based on a list of predetermined keywords (exact search queries depicted in **Table 1**), with no temporal restrictions and no other applied filter.

**Table 1:** Keyword combinations used in queries in both PubMed and Scopus, alongside the number of results produced in the initial search.

Database	Search query	No of results PubMed + Scopus = Total results (no deduplication)
PubMed+Scopus	(“psychotic” AND “relapse” AND “prediction”)	454 + 181 = 635
PubMed+Scopus	(“psychotic” AND “relapse” AND “biomarkers”)	64 + 40 = 104
PubMed+Scopus	(“schizophrenia” AND “relapse” AND “prediction”)	633 + 299 = 932
PubMed+Scopus	(“schizophrenia” AND “relapse” AND “biomarkers”)	121 + 99 = 220
		Results across all queries:1891

After removing duplicate records, we scanned (AS and CT) the titles and abstracts of the retrieved studies independently and excluded those that were irrelevant to the research question. Any discrepancies were addressed by a third independent reviewer (PF or NS). The main reason for exclusions at this stage of the process was that in search queries using the key word “prediction”, which yielded more results, the utilization of biomarkers instead of clinical variables as predictors was relatively rare. We then defined (see **Table 2** below) and applied the PICOS/PECOS worksheet criteria to a pool of 121 full papers, ultimately selecting a total of 42 studies for inclusion.

**Table 2:** Inclusion and exclusion criteria designed according to the blueprint of the Population, Intervention or Exposure, Comparison, Outcomes, and Study Design (PICOS/PECOS) worksheet.

Parameter	Inclusion criteria	Exclusion criteria
Participants	Patients experiencing symptom relapse	Population of solely FEP patients
Interventions or Exposures	Measurements of biomarkers, in the genetic, blood-based, neuroimaging, cognitive or behavioral domain	Solely clinical assessments, effects of different treatment regimens.
Comparisons	Within the patient group at different time points for longitudinal studies, relapsed vs healthy controls or vs FEP for cross-sectional studies	Treatment groups, groups with different diagnoses, or groups consisting of solely FEP patients in cross-sectional comparisons.
Outcomes	Relapse prediction, or relapse risk assessment	Diagnosis distinction, response to medication
Study Design	Original studies in English, longitudinal or cross sectional, if and only if the patient group consisted of relapsed patients	Reviews either narrative or systematic, meta-analyses.

Since the goal of the present review is to assess available means for relapse prediction via biomarker monitoring, we only included original studies using biological or behavioral factors, involving relapsed patients. The notion of prediction implies monitoring the evolution of some phenomenon in time and drawing conclusions regarding occurrence rate and time of onset relative to some fixed time point. This would suggest that only longitudinal studies should be included, however, we also include cross sectional studies where patients experiencing relapse are compared to FEP patients or healthy controls. While this design introduces a plethora of confounding factors (for instance FEP patients are usually drug-naïve, whereas relapsed patients have taken or are still taking antipsychotic medication), it is still meaningful to consider them, since for certain categories, such as blood-based biomarkers, continuous and even sequential monitoring presents extreme practical difficulties. Regarding study outcomes, we only included studies where the main objective was to either predict relapse in a temporal sense, or to at least shape an *a priori* relative-risk profile. Studies related to diagnosis or treatment effects were excluded.

The 42 studies that were finally selected, were thoroughly analyzed, and the following information was extracted: Authors and country of origin, Study design (longitudinal or cross sectional), Sample size and diagnosis for patient groups, Data collection process, examined biomarkers, Analysis tools, Main objectives related to relapse, Statistical results and Synthesis of main findings. Data extraction was initially performed by AS, and was independently validated by CT, PF, and NS. No protocol was registered beforehand for this review. Excel files and data used in this systematic review are available upon request.

### 2.4 Quality assessment of included studies

The Appraisal tool for Cross-Sectional Studies (AXIS (17), **Table 3**) was utilized for risk of bias assessment of included studies and it encompasses questions related to study objective and design, sample size justification, sample selection process and reasons for exclusion of participants, internal validity, presentation and replicability of statistical tools and their corresponding results as well as limitations, funding sources or conflicting interests. Note that although theoretically the AXIS tool is tailored to cross sectional studies, almost all questions can apply to a wider range of study designs. Reviewers had the same assigned roles as in the data extraction procedure.

**Table 3:** Quality assessment of included studies via the appraisal tool for cross-sectional studies (AXIS).

The Appraisal tool for Cross-Sectional Studies (AXIS) study (Y = YES, N = NO, - = NOT APPROPRIATE)																					
Study	(21)	(22)	(23)	(24)	(25)	(26)	(27)	(28)	(29)	(30)	(31)	(32)	(33)	(34)	(35)	(36)	(37)	(38)	(39)	(40)	(41)
Introduction																					
1. Were the aims/objectives of the study clear?	Y	Y	Y	Y	Y	Y	Y	Y	Y	Y	N	Y	Y	Y	Y	Y	Y	Y	Y	Y	Y
Methods																					
2. Was the study design appropriate for the stated aim(s)?	Y	Y	Y	Y	N	N	N	Y	Y	Y	Y	N	Y	Y	Y	N	Y	N	Y	Y	Y
3. Was the sample size justified?	N	N	N	N	N	N	N	N	N	Y	N	Y	N	N	N	N	N	N	N	N	N
4. Was the target/reference population clearly defined?	Y	Y	Y	Y	Y	N	Y	Y	Y	Y	Y	N	Y	Y	Y	Y	Y	Y	Y	Y	Y
5. Was the sample frame taken from an appropriate population base so that it closely represented the target/reference population under investigation?	Y	Y	Y	Y	N	Y	Y	Y	N	Y	Y	N	N	Y	N	Y	Y	Y	N	Y	Y
6. Was the selection process likely to select subjects/participants that were representative of the target/reference population under investigation?	Y	Y	Y	Y	N	Y	Y	Y	N	Y	Y	N	N	Y	N	N	Y	N	N	Y	Y
7. Were measures undertaken to address and categorize Nn-responders?	N	N	N	N	N	N	N	Y	N	N	N	Y	N	Y	Y	N	N	N	N	N	Y
8. Were the risk factor and outcome variables measured appropriate to the aims of the study?	Y	Y	Y	Y	Y	Y	Y	Y	Y	Y	Y	Y	Y	Y	Y	Y	Y	Y	Y	Y	Y
9. Were the risk factor and outcome variables measured correctly using instruments/measurements that had been trialled, piloted or published previously?	Y	Y	Y	Y	Y	Y	Y	Y	Y	Y	Y	N	Y	Y	Y	Y	Y	Y	Y	Y	Y
10. Is it clear what was used to determined statistical significance and/or precision estimates? (eg, p values, CIs)	Y	Y	Y	Y	Y	Y	Y	Y	Y	Y	Y	Y	Y	Y	Y	Y	Y	Y	Y	Y	Y
11. Were the methods (including statistical methods) sufficiently described to enable them to be repeated?	N	Y	Y	Y	Y	Y	Y	Y	Y	Y	N	N	Y	N	Y	Y	Y	N	Y	Y	Y
Results																					
12. Were the basic data adequately described?	Y	Y	Y	Y	Y	Y	Y	Y	Y	Y	Y	Y	Y	Y	Y	Y	Y	Y	Y	Y	Y
13. Does the response rate raise concerns about Nn-response bias?	N	N	Y	N	N	N	N	N	N	N	N	N	N	Y	Y	N	Y	N	N	Y	N
14. If appropriate, was information about Nn-responders described?	–	–	N	–	–	–	–	–	–	–	–	–	–	Y	Y	–	N	–	–	N	–
15. Were the results internally consistent?	Y	Y	Y	Y	Y	Y	Y	Y	Y	Y	Y	Y	Y	Y	Y	Y	Y	Y	Y	Y	Y
16. Were the results for the analyses described in the methods, presented?	Y	Y	Y	N	Y	Y	Y	Y	Y	Y	Y	Y	Y	Y	Y	Y	Y	Y	Y	Y	Y
Discussion																					
17. Were the authors’ discussions and conclusions justified by results?	Y	N	Y	Y	Y	N	N	Y	N	Y	Y	Y	Y	Y	N	N	Y	Y	Y	Y	Y
18. Were the limitations of the study discussed?	Y	Y	Y	Y	Y	Y	Y	Y	Y	Y	Y	Y	Y	Y	Y	Y	Y	Y	Y	Y	Y
Other																					
19. Were there any funding sources or conflicts of interest that may affect the authors’ interpretation of results?	N	N	Y	Y	N	Y	Y	Y	N	Y	Y	N	Y	Y	N	N	Y	Y	Y	N	Y
20. Was ethical approval or consent attained?	Y	Y	Y	Y	Y	Y	Y	Y	Y	Y	Y	Y	Y	Y	Y	Y	Y	Y	Y	Y	Y
Introduction																					
1. Were the aims/objectives of the study clear?	Y	Y	Y	Y	Y	Y	Y	Y	Y	Y	Y	Y	Y	Y	Y	Y	Y	Y	Y	Y	Y
Methods																					
2. Was the study design appropriate for the stated aim(s)?	N	Y	Y	N	Y	N	N	N	Y	Y	Y	Y	Y	Y	Y	Y	Y	Y	Y	Y	Y
3. Was the sample size justified?	N	N	N	N	Y	N	N	N	N	N	N	N	N	N	N	N	N	N	N	N	N
4. Was the target/reference population clearly defined?	Y	Y	Y	Y	Y	Y	Y	Y	Y	Y	Y	Y	Y	Y	Y	Y	Y	Y	Y	Y	Y
5. Was the sample frame taken from an appropriate population base so that it closely represented the target/reference population under investigation?	Y	Y	N	Y	Y	Y	Y	Y	Y	Y	Y	Y	N	Y	N	Y	Y	Y	Y	Y	Y
6. Was the selection process likely to select subjects/participants that were representative of the target/reference population under investigation?	N	Y	N	Y	Y	Y	Y	N	Y	Y	Y	Y	N	Y	N	Y	Y	Y	Y	Y	N
7. Were measures undertaken to address and categorize Nn-responders?	N	Y	N	Y	N	N	N	N	N	Y	N	N	N	N	N	N	N	N	N	N	N
8. Were the risk factor and outcome variables measured appropriate to the aims of the study?	N	Y	Y	Y	Y	Y	Y	Y	Y	Y	Y	Y	Y	Y	Y	Y	Y	Y	Y	Y	Y
9. Were the risk factor and outcome variables measured correctly using instruments/measurements that had been trialled, piloted or published previously?	Y	Y	Y	Y	Y	Y	Y	Y	Y	Y	N	N	Y	N	Y	N	N	N	Y	Y	Y
10. Is it clear what was used to determined statistical significance and/or precision estimates? (eg, p values, CIs)	Y	Y	Y	Y	Y	Y	Y	Y	Y	Y	Y	Y	Y	N	Y	Y	Y	Y	Y	Y	Y
11. Were the methods (including statistical methods) sufficiently described to enable them to be repeated?	Y	Y	Y	Y	Y	Y	Y	Y	Y	Y	Y	Y	Y	N	Y	Y	Y	Y	Y	Y	Y
Results																					
12. Were the basic data adequately described?	Y	Y	Y	Y	Y	Y	Y	Y	Y	Y	Y	Y	Y	Y	Y	Y	Y	Y	Y	Y	N
13. Does the response rate raise concerns about Nn-response bias?	N	Y	N	N	N	N	N	N	Y	Y	N	N	N	N	N	Y	N	N	Y	N	N
14. If appropriate, was information about Nn-responders described?	–	Y	–	–	–	–	–	–	N	Y	–	–	–	–	–	N	–	–	N	–	–
15. Were the results internally consistent?	Y	Y	Y	Y	Y	Y	Y	Y	Y	Y	Y	Y	Y	Y	Y	Y	Y	N	Y	Y	N
16. Were the results for the analyses described in the methods, presented?	Y	Y	Y	Y	Y	Y	Y	Y	Y	Y	Y	Y	Y	N	N	Y	Y	Y	Y	Y	N
Discussion																					
17. Were the authors’ discussions and conclusions justified by results?	Y	N	Y	N	N	Y	N	Y	Y	Y	N	Y	Y	Y	Y	N	Y	N	Y	Y	Y
18. Were the limitations of the study discussed?	Y	Y	Y	Y	Y	Y	Y	Y	N	Y	Y	Y	Y	Y	Y	Y	Y	Y	Y	Y	Y
Other																					
19. Were there any funding sources or conflicts of interest that may affect the authors’ interpretation of results?	Y	Y	Y	Y	Y	Y	Y	Y	Y	Y	Y	Y	Y	Y	Y	Y	Y	Y	Y	Y	Y
20. Was ethical approval or consent attained?	Y	Y	Y	Y	Y	Y	Y	Y	Y	Y	Y	Y	Y	Y	Y	Y	Y	Y	Y	Y	Y

## 3 Results

**Figure 1:**
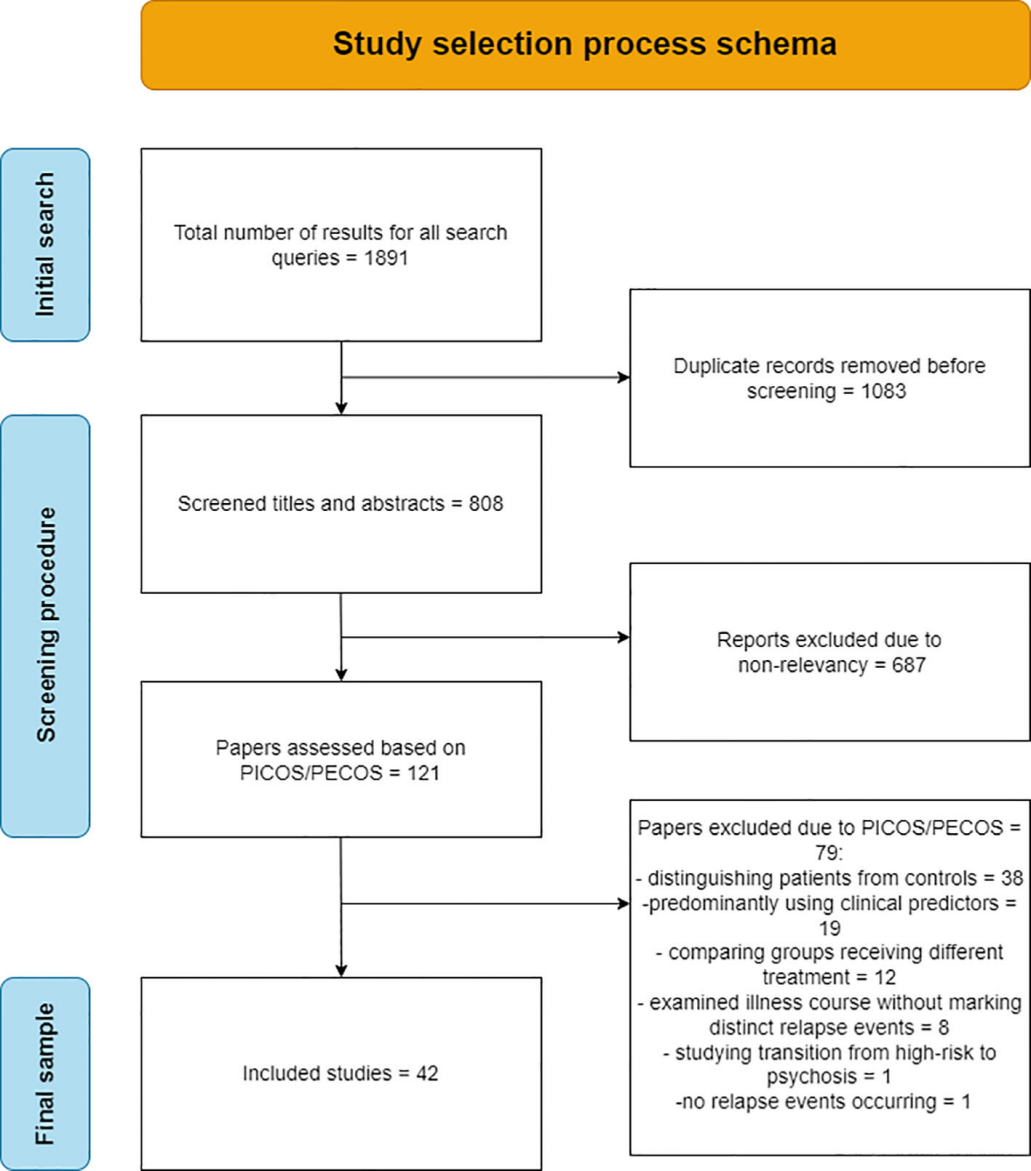
Flowchart of the selection process of the final 42 papers analyzed in the review.

**Figure 1** depicts a schematic representation of the selection process, which starts with a series of queries in the PubMed and Scopus databases yielding a total of 1891 results. Given the conceptual closeness of the various keyword combinations, duplicate records were expected and after their removal, a list of 808 unique papers was compiled. Of these 808 papers, 687 were excluded from the title and abstract, on grounds of non-relevancy to the research question. After application of the defined PICOS/PECOS criteria to the remaining 121 papers, a total of 42 were finally selected and analyzed. Of the 79 papers excluded based on PICOS/PECOS, 19 incorporated models using predominantly clinical parameters and not biomarkers, 38 were focused on distinguishing patients from controls, 12 made comparisons between groups receiving different treatment, 8 examined illness course via symptom scales such as PANNS, but relapse events were not identified and classified, 1 (18) sought to predict relapse, but no relapse instances occurred due to limited study duration and 1 (19) revolved around the transition of patients from high-risk to psychosis.

The final 42 studies were grouped based on the nature of the examined biomarkers. This categorization consisted of:

1. the genetic biomarker subgroup (n = 4, or 9%) summarized in **Table 4**,
2. the blood-based biomarker subgroup (n = 15, or 36%) summarized in **Table 5**,
3. the neuroimaging biomarker subgroup (n = 10, or 24%) including studies in structural Magnetic Resonance Imaging (MRI), functional MRI, electroencephalogram (EEG) and Positron Emission Tomography. summarized in **Table 6**,
4. the cognitive-behavioral biomarker subgroup (n = 5, or 12%) including markers used to assess performance in cognitive domains such as memory, attention, perception and executive function, as well as behavior markers based on internet search history and Facebook posting habits, summarized in **Table 7**,
5. and the wearables biomarker subgroup (n = 8, or 19%). which encompasses studies that apply machine learning models on passively collected data from wearable devices such as smartwatches, but also from smartphones, to identify sudden pattern breaks constituting the signature of impending relapse, summarized in **Table 8**.

**Table 4:** Presentation of analysis results for papers using genetic biomarkers.

Study/Country	Study Design	Sample (Diagnosis)	Data Collection Process	Biomar-kers examined	Analysis Tools	Main objectives	Statistical Results	Synthesis of main findings	
Meier et al.2015 (21) (Denmark/Germany)	Cross sectional	n = 1681 patients SCZ, ICD-10 for Danish sample/n = 1306 SCZ for German sample	DNA extracted from dry blood spots and genome-wide amplified	>Genomic risk profile score (GPRS), consisting of alleles at thou-sands of loci	Logistic regression model for GPRS calculation, Spearman’s rank correlation to assess relationship between GPRS and number of admissions	>Identify a link between GPRS and admission frequency/duration	Danish Sample: Patients divided into 4 groups (SCZ or other, in or out-patient). Analysis was repeated with different P value thresholds (0.05,0.1,0.2) for inclusion of single nucleotide polymorphisms. For a P-value threshold of 0.05, Spearman’s r values for GPRS with number of admissions were 0.053, 0.063, 0.044, 0.054 and the corresponding P-values were 0.066, 0.037, 0.103, and 0.06. German Sample: Spearman’s r = 0.066 with a P-value of 0.016. For a P-value threshold of 0.2 (21) report the following r values (0.077,0.081,0.074,0.073) with the corresponding P-values (0.014,0.01,0.016,0.018) for the Danish sample, and an r = 0.062 with P =0.024 for the German sample.	GPRS correlates with frequency and duration of admissions. The highest correlation values were observed when the threshold for including single-nucleotide polymorphisms was set to 0.2, when compared to lower values, implying that chronic SCZ could be associated with a wider range of susceptibility variants.	
Pawelczyk et al., 2015 (22) (Poland)	Cross sectional	n = 86 patients with SCZ according to ICD-10 criteria (Non-random, convenience sample), who were split into two groups: Early-SCZ, n=42 and Chronic SCZ, n=44. The criterion for classification in the chronic group, was one distinct relapse event and a minimum of 2 years from illness onset.	Genomic DNA from blood samples, telomeric DNA amplified via qPCR assay. Clinical evaluation using PANNS for SCZ symptoms and GAF for overall psychoso-cial functioning	>Telomere length	A one-way Analysis of Covariance (ANCOVA) was utilized to control for confounders, mainly age, with PANNS scores and hospital admissions/number of psychotic episodes as the dependent variables. Univariate analyses (Spearman’s rank correlation) were performed to examine correlations of telomere length with disease severity/chronicity irrespective of group.	>Assessing whether shortening of telomere length predicts disease severity and number of relapses	In univariate analyses, telomere length negatively correlated with number of psychotic episodes (r = -0.594/p <0.001). Subgroup analyses presented the same trends but did not reach significance. In the ANCOVA, a difference in telomere length between patients with E-SCZ and C-SCZ (F [1,82] =47.08, P<0.001) was reported.	Telomere length is connected to disease chronicity, since a (negative) correlation was found between TL and number of psychotic episodes. Furthermore, telomere length was shorter in patients with the Chronic Subphenotype, indicating that telomere erosion could lead to a more severe disease course.	
Gasso et al., 2021 (23) (Spain)	Prospective (2 samples: at baseline and after 3 years or at relapse)	n= 91 patients at baseline, n = 31 at relapse, n = 36 after 3 years of follow up. SCZ diagnosis according to DSM-IV, all patients in remission at baseline, according to Andeassen’s criteria (20).	Total RNA (Clariom S Human Array) covers over 337,100 transcripts and variants, which in turn represent 20,800 genes.) from blood samples, clinical follow-up every 3 months, for 3 years, via PANNS for symptomatology	>25 modules or “network” type structures of co-expres-sed genes. Module sizes (in number of genes) varied from 41 to 5627.	Genome-wide expression analysis to construct the modules or “networks” of co-expressed genes. Preservation analysis to examine longitudinal changes to these modules. Receiver operating characteristic (ROC) curve analysis [] to assess the predictive properties of selected modules. Kaplan–Meier and Cox regression analysis to assess the effect of the best performing network on time taken to relapse.	>To examine whether changes in network connectivity properties of co-expressed gene clusters can predict relapse.	DarkRed module consisting of 53 genes (alongside DarkGrey, 41 genes) were semi-conserved (i.e. Zsummary statistic > 2 but <10. The Zsummary statistic is used to assess similarity in connectivity patterns between two networks, with higher values indicating more similar, persisting patterns) after 3 years of follow-up and at relapse. Relapse prediction accuracy of the semi-conserved modules was tested with ROC curve analysis. DarkRed module had the highest predictive value (AUC = 0.603, CI = 0.464-0.742), followed by Dark-Grey (AUC = 0.556, CI = 0.414–0.699), but all p values > 0.05. Kaplan–Meier and Cox regression analysis: Patients were split based on gene expression for DarkRed module. Those at the highest 75 percentile (N = 20) showed higher risk of suffering a relapse (OR = 2.10, CI = 1.01–4.33,beta = 0.742 ± 0.370, p = 0.045).	The ability of semi-conserved networks of co-expressed genes to predict relapse did not reach the significance threshold. However, patients with higher expression of genes in the DarkRed module showed higher risk of relapse and earlier appearance of relapse. DarkRed module genes participate in biological processes related to the ubiquitin proteosome system, which influences neuronal development.	
Segura et al.2023 (24) (Spain)	Cross sectional	n = 114 patients (SCZ, DSM-IV)/ 58 relapse, 56 non-relapse	Genomic DNA from blood samples, clinical follow-up every 3 months, for 3 years, via PANNS for symptomatology	>Polygenic risk score	Binary logistic regression, with relapse as the dependent variable and polygenic risk score as the independent variable. (covariates: sex, age, ethnicity and the first 10 components of the genetic Principal Components Analysis)	>Find group differences in Polygenic risk scores between relapsers and non-relapsers.	4 groups for High Polygenic Risk (for Schizophrenia, Bipolar Disorder, educational attainment, cognitive performance). At the p= 0.05 threshold for selecting risk alleles, there was a significant association with relapse risk only in the Educational Attainment group. (β = -0.042, p = 0.043), with an R squared value of 2.1%.	For high-risk SCZ and BP groups, there was no significant association between Polygenic Risk Score and relapse, for any of the p threshold values for selecting risk alleles.

**Table 5:** Presentation of synthesized findings from papers using non-genetic, blood-based biomarkers.

Study/Country	Study Design	Sample (Diagnosis)	Data Collection Process	Biomar-kers examined	Analysis Tools	Main objectives	Statistical Results	Synthesis of main findings	
Kaddurah-Daouk et al., 2012 (25) (USA)	Cross sectional	n = 40 patients (SCZ, DSM-IV)/20 drug-naive FEP, 20 acutely relapsed	Blood samples, assessment of plasma lipid profiles. BPRS for clinical symptom severity.	>5 different human plasma phospholi-pids	Wilcoxon rank-sum test to assess differences between patients and controls, as well as FEP and relapsed patients. False discovery rate was used to correct for multiple comparisons.	>Comparing different plasmalogens concentrations in FEP vs relapsed patients.	There were no significant differences in any of the examined plasmalogens (Phosphatidylethanolamine class, containing fatty acids 16:0, 18:0, 18:1n7, 18:1n9/Phosphatidylcholine class, containing fatty acids 16:0, 18:0, 18:1n7, 18:1n9). Statistical data was not shown in the paper, as the main focus was the comparison between all patients combined and healthy controls.	Plasmalogens correlate with diagnosis of SCZ, but no significant differences were observed between FEP and relapsed patients, indicating that they are probably not appropriate biomarkers to predict chronic disease course.	
Schwartz et al.2012 (26) (Germany)	Prospective	n = 77 patients (DSM-IV SCZ)/18 experienced relapse, 59 did not. Patients who relapsed were further classified into two groups, each consisting of 9 subjects, depending on the time elapsed between last clinical visit and relapse. (short-term relapse vs long-term relapse)	Blood samples at baseline (acute phase), after 6 weeks of treatment, and during relapse (for patients who relapsed). Multiplexed immuno-assays to measure serum concentra-tions of 191 proteins and small molecules. Clinical assessment with PANNS.	>191 proteins and small molecules	Shapiro-wilk test to test normality of each analyte’s distribution. Associations were examined with non-parametric Spearman’s correlations, adjusted for false discovery rate. Group comparisons (short-term relapse vs long-term relapse) were performed with the Wilcoxon rank sum test. For classification of patients in the relapse or non-relapse group based on the values of serum molecules, a Random Forests analysis was utilized.	>Identify predictors of time to relapse among a panel of 191 candidate molecules.	Random forests analysis lead to the selection of a group of molecules comprised of leptin,proinsulin, TGF-a, b-cellulin, CD5L, CD40, Apo CI, clusterin, insulin, interleukin-8, MIP-1-b and matrix metalloproteinase 3, whose combination predicted short vs long term relapse at an accuracy of 94.5%. BMI changes alone achieved a predictive accuracy of 83.4%. Non-parametric Wilcoxon tests for each molecule, resulted in significant differences for 13 of those after performing ANCOVA (on log10 transformed data) to account for the effect of BMI. Those were Leptin (p = 0.033), Proinsulin (p = 0.021), Transforming growth factor alpha (p = 0.018), b-Cellulin (p = 0.015), CD5 L (p = 0.002), Matrix metalloproteinase 9 (p = 0.029), CD40 (p = 0.011), Macrophage-derived chemokine (p = 0.021), Brain-derived neurotrophic factor (p = 0.042), Apolipoprotein CI (p = 0.046), Matrix metalloproteinase 7 (p = 0.045), b-2–Microglobulin (p = 0.046) and Tumor necrosis factor receptor like 2 (p = 0.039).	Alterations in the concentrations of certain serum molecules may be predictive of the time to relapse. However, antipsychotic treatment (or non-compliance to it), remains a significant confounding factor, especially for molecules related to metabolism, such as leptin, pro-insulin, insulin and C-peptide.	
Borovcan-in et al., 2015 (27) (Serbia)	Cross sectional	n = 125 patients: 78 FEP (F23 according to ICD-10) and 47 acutely relapsed (F20 according to ICD-10)	Blood samples and ELISA immunoassay to determine IL-23 levels. Clinical assessment with PANNS.	>IL-23	Shapiro-wilk test to assess normality of IL-23 distribution. Kruskal-Wallis test was used to evaluate group differences in IL-23 levels.	>Comparing IL-23 levels between FEP patients, relapsed patients, and healthy controls.	There was no significant difference in serum IL-23 levels between FEP and relapsed patients. (511.70 ± 66.25 for FEP, 652.08 ± 119.24 for relapse, p = 0.63) No changes for FEP or relapsed patients in IL-23 levels after 4 weeks of treatment (p = 0.656 and p = 0.706).	IL-23 could be a useful trait marker for SCZ, since they differ between SCZ patients and HC, but not a state marker, since there were no differences in IL-23 levels between FEP patients and relapsed patients.	
Morera-Fumero et al., 2017 (28) (Spain)	Prospective (samples at admission, and at discharge)	n = 43 SCZ patients (DSM-IV)	Blood samples collected at admission-discharge. Total Antioxidant Capacity (TAC) was measured, which is the antioxidant capacity of water soluble molecules such as albumin or transferrin. Clinical Global Impressions (CGI) scale for clinical symptoms.	>Total Antioxidant Capacity (TAC)	ANOVA for repeated measures to compare group mean at admission and discharge.	>Comparing TAC values during acute psychotic relapse and after remission in the same group of patients.	TAC values did not differ significantly between admission and discharge (mean values: 0.66+/- 0.14 and 0.64+/- 0.15, p > 0.05)	While patients have significantly lower TAC values than controls, both at admission and discharge, the difference within the patient group at the two time points did not reach significance.
Szymona et al.2017 (29) (Poland)	Prospective (2 samples, during relapse and remission)	n = 51 patients (SCZ, ICD-10)	Blood samples during relapse then at remission, determined by PANNS. EDTA tubes were used for plasma cytokines analysis and clot activating tubes were used for serum analyses of kynurenines.	>Kynurenic Acid (KYNA), 3-Hydroxykynurenine (3-HK), sIL-2R, IFN-α, IL-4	Mann-Whitney U test for comparisons between the same group of patients at relapse and at remission.	>Assessing levels of kynurenic acid, 3-hydroxyky-nurenine, and various cytokines during relapse and remission	There were no significant differences between relapse and remission in any of the examined biomarkers. Mann-Whitney U test results for KYNA measured in pmol/100μl (relapse: 0.95 ± 0.45, remission: 0.97 ± 0.33, p > 0.05), for 3HK in pmol/100μl (relapse: 8.54 ± 4.19, remission: 10.1 ± 6.75, p > 0.05), for sIL-2R in ng/ml (relapse: 0.88 ± 0.46, remission: 0.88 ± 0.32, p > 0.05), for IFN-α in pg/ml (relapse: 13.1 ± 22.66, remission: 9.14 ± 15.64, p > 0.05).	While KYNA, 3HK and IL-4 levels differed between patients and controls, and thus have potential as trait markers, none of the examined substances differed between relapse and remission, indicating that they are not likely candidates as state markers for acute relapse.
Pillai et al., 2017 (30) (USA)	Prospective (maximum 23 samples per subject over a period of 30 months)	n = 221 patients with SCZ or SAD according to DSM-IV	Blood sample every 6 weeks or at relapse, BDNF assessed with ELISA. Clinical follow up for 30 months.	>plasma BDNF	ROC curve analysis to test the predictive value of baseline BDNF for relapse. Cox regression to model time to relapse as a function of baseline BDNF. Linear regression of successive BDNF values on visit number was performed to measure rate of change, which, alongside summary statistics such as per subject mean, mode, and variance was compared in the group of relapsers vs non-relapsers, via the Wilcoxon rank sum test.	>Correlating baseline BDNF and BDNF variation in time with the occurre-nce of relapse.	ROC curve analysis yielded negative results for BDNF as a predictor of relapse.(AUC = 0.495, p = 0.901 when comparing with the minimally accepted AUC value) Cox regression did not associate baseline BDNF with risk of relapse(Hazard Ratio = 1.000,95% CI = 0.998 - 1.001, p = 0.717) Mean per subject BDNF values did not differ between the group of relapsers and non-relapsers.(p = 0.893)	Although BDNF has been found to correlate with diagnosis of SCZ, it does not seem to have predictive value for adverse outcomes throughout the course of the disease. In a large sample which was prospectively followed up on, neither baseline BDNF values, nor their average over a 30 month time period, could distinguish between the group of patients who subsequently relapsed and the group of those who did not.
Piotrowski et al., 2019 (31) (Poland)	Cross sectional	n = 67 patients (DSM-IV SCZ, SAF, Schizophre-niform disorder, Brief psychotic episode) with 42 FEP patients and 25 acutely relapsed patients	Fasting blood samples, calculation of biochemical parameters in serum. Weight, height, hip and waste circumfe-rence measure-ments. PANNS, GAF, Social and Functioning Assessment Scale (SOFAS) were used for clinical assessment.	>Allostatic (AL) Index, encompassing Systolic and Diastolic blood pressure, BMI, waste to hip ratio, high sensitivity CRP, fibrinogen, albumin, fasting glucose and insulin, total cholesteroltriglyce-rides and cortisol.	In normally distributed variables, ANOVA was used to test for group differences, whereas in non-normally distributed variables, the Mann-Whitney U test was utilized instead. For group differences in the AL index, ANCOVA was used, with age, sex and smoking status as covariates.	>Comparing the level of biological dysfuncti-on, captured by an index comprised of 15 different biomarkers, in FEP and acutely relapsed patients.	ANCOVA resulted in a significant difference in AL index between FEP and acutely relapsed patients (F = 7.99, p < 0.001). Regarding single biomarkers there were significant differences in Systolic Blood Pressure (FEP: 120.2 ± 11.2, relapse: 127.8 ± 8.3, p = 0.001), Waste to Hip Ratio (FEP: 0.85 ± 0.10, relapse: 0.90 ± 0.09, p= 0.003), hsCRP (FEP: 1.2 ± 1.9,relapse: 3.4 ± 3.3, p = 0.006), insulin (FEP: 22.7 ± 22.3, relapse: 15.9 ± 13.5, p = 0.019) and cortisol (FEP: 334.2 ± 74.6, relapse: 443.8 ± 146.7, p < 0.001).	Chronic SCZ patients present more severe systemic biological dysregulations, captured by the AL index, compared to FEP patients. This could be due to continuously elevated stress levels, consistent smoking and poor dietary habits, as well as antipsychotic treatment side effects.
Ozdin and Boke (32) (Turkey)	Retrospe-ctive (2 samples, during relapse and remission)	n = 105 patients (SCZ, DSM-IV)	Blood samples were collected during relapse and then at remission, determined by PANNS scores. Calculation of white blood count (WBC), neutrophils, platelets, lymphocytes, monocytes, as well as the neutrophil to lymphocyte ratio (NLR), platelet to lymphocyte ratio (PLR) and monocyte to lymphocyte ratio (MLR) was performed.	>NLR, PLR, MLR, WBC, Neutro-phils, Platelets, Lympho-cytes, Monocytes	Wilcoxon rank-sum test to assess differences between the same patients at relapse and at remission. No reported correction for multiple comparisons.	>Identify differences in ratios of complete blood count markers, between relapse and remission in the same group of patients.	Wilcoxon rank sum tests yielded the following results, for patients at relapse and at remission. For the NLR: mean at relapse (interquartile range in parentheses): 119.80 (1.47), mean at remission: 91.20 (1.37), Z = −3.90, p < 0.01. For the PLR: mean at relapse: 112.84 (63.70), mean at remission: 98.16 (50.98), Z = −2.35, p = 0.019. For the MLR: mean at relapse: 121.01 (0.11), mean at remission: 89.99 (0.08), Z = −4.58, p < 0.01. For the WBC: mean at relapse: 115.68 (3.28), mean at remission: 95.32 (2.51), Z = −3.21, p = 0.001. For Neutrophils: mean at relapse: 119.04 (2.62), mean at remission: 91.96 (1.72), Z = −3.81, p < 0.01. For Platelets: mean at relapse: 110.17 (90.50), mean at remission: 100.83 (48.50), Z = −2.08, p = 0.033. For Monocytes: mean at relapse: 116.51 (0.22), mean at remission:94.49 (0.17), Z = −3.24, p = 0.001. For Lymphocytes: mean at relapse: 101.92 (0.84), mean at remission:109.08 (0.70), Z = −1.46, p = 0.143.	Multiple markers (with the exception of Lymphocytes), indicative of inflammation, originating from the complete blood count, were found to be significantly elevated during relapse compared to remission. These differences could be due to continuation of antipsychotic treatment.
Luo et al.2019 (33) (China)	Prospective (2 samples, at admission and at discharge)	n = 68 patients (ICD-10 SCZ)	Fasting blood samples at admission and discharge, ELISA immunoassay to measure TNF-α, IL-6 and IL-18 concentrations. PANNS to assess clinical symptomatology.	>TNF-α, IL-6, IL-18	Paired t-test to test for differences in cytokine concentrations between admission and discharge for the same group of patients.	>Assessing TNF-α, IL-6 and IL-18 as potential trait markers for SCZ, as well as state markers for relapse.	Paired t-tests for patients at admission and patients at relapse yielded the following (cytokines measured in pg/ml): for TNF-α (admission 12.15 ± 4.01, discharge 11.30 ± 3.66, p > 0.05) for IL-18 (admission 73.60 ± 13.92, discharge 68.47 ± 13.31, p > 0.05) and for IL- 6 (admission 5.61 ± 1.97, discharge 1.62 ± 0.19, p = 0.001)	TNF-α, IL-6 and IL-18 have been found to be elevated in SCZ patients when compared to controls, which was corroborated in this study. Only IL-6 differed significantly at discharge when compared to admission, potentially highlighting IL-6 as a state marker for relapse.
Martínez-Pinteño et al., 2022 (34) (Spain)	Prospective (2 samples: at baseline and after 3 years or at relapse	n = 69 patients (DSM-IV SCZ spectrum)/32 relapse - 37 non-relapse	peripheral blood sample, BDNF and NGF (Nerve growth factor) assessed with ELISA. Clinical follow up every 3 months for 3 years, via PANNS for symptoma- tology	>plasma BDNF and NGF	ROC curve analysis to test the predictive value of baseline BDNF and NGF for relapse. Mann-Whitney U test to assess differences between baseline and follow up BDNF/NGF levels. These differences were then correlated with relapse classification (between subjects variable), using general linear models with repetitive measures and time as the within subject factor	>Correlating longitudi-nal variation of BDNF/NGF with relapse classificati-on.	ROC curve analysis yielded negative results for BDNF/NGF as predictors of relapse. (AUC = 0.473, p = 0.0698 for BDNF and AUC = 0.444, p = 0.931 for NGF) Longitudinal differences between relapse and non-relapse groups did not differ significantly. (F = 2.339, p-value=0.131 for BDNF and F = 2.633, p-value=0.110 for NGF)	Neither BDNF nor NGF differences (relapse- baseline) correlated with relapse classification. Furthermore, in the relapse group, no differences were observed between baseline and relapse BDNF/NGF values, indicating that BDNF/NGF are unlikely to be suitable as biomarkers for psychotic relapse prediction.
Marques and Ouakinin 2022 (35) (Portugal)	Prospective (2 samples: first during relapse, then at remission)	n = 60 patients: 30 SCZ, 30 SAF according to ICD-10)	Fasting blood samples and calculation of Unconju-gated Bilirubin (UCB = total Bilirubin - direct bilirubin). PANNS for assessment of psychotic symptoma-tology/PSP for social functioning.	>Unconju-gated bilirubin	At relapse: ANOVA with Bonferroni correction for multiple comparisons, to evaluate differences between the three groups. Same process for remission. Same analysis was also utilized to compare relapse and remission within each group.	>Assessing UCB as a biomarker for SCZ and SAF, both at relapse and at remission.	At relapse, UCB levels differed significantly for both SCZ and SAF, when compared to the Bipolar Disorder group, utilized as a control group. (p < 0.05, no F-Statistic reported) At remission, the analysis yielded the same results, with an added difference between SCZ and SAF. (p = 0.05, no F-Statistic) Regarding group differences between relapse and remission, there was a decrease, which did not reach significance for the SCZ group (p = 0.05, no F-Statistic) but did for the SAF group (p = 0.34, no F-Statistic).	Unconjugated bilirubin was found to differ significantly between SCZ or SAF and BP patients, both during relapse and during remission. Furthermore, UCB could serve as a biomarker for relapse, due to the difference reported within each group between relapse and remission.
Fabrazzo et al., 2022 (36) (Italy)	Cross sectional	n = 152 patients with psychiatric diagnosis (SCZ, SAF, OCD, BP) according to DSM-V. 74 during acute relapse, 78 stable out-patients.	Blood samples and measure-ment of 25-OH Vitamin D and Parathyroid Hormone (PTH) via chemilumi-nescence immuno-assays. BPRS for symptom severity.	>Vit-D, PTH	Students t-test for independent samples was utilized with serum levels of PTH, 25-OH-VitD and calcium by patient status (relapsed inpatient vs stable outpatients	>Comparing Vit-D, PTH and calcium levels of acutely relapsed patients with stable outpatients	Vit-D for relapsed vs stable outpatients respectively: 23.1 ± 13.4 pg/mL and 28.3 ± 15.0 pg/mL (p < 0.001, no t-statistic reported) PTH same comparison: 30.6 ± 20.1 pg/mL and 38.5 ± 24.4 pg/mL (p < 0.001, no t-statistic) Calcium same comparison: 9.3 ± 6.4 mg/dL and 9.2 ± 0.8 mg/dL, no group difference.	Significant differences in serum Vit-D and PTH between in-patients experiencing acute relapse and stable outpatients, could implicate deficiencies of these two biomarkers in the pathogenesis of relapse.
Miller et al., 2023 (37) (USA)	Prospective (blood samples up to every 3 weeks, for 30 months)	n = 200 patients (SCZ)/70 relapse, 130 non-relapse	Blood samples collected up to every 3 weeks for the 30-month duration of the study. (mean number of samples was 10.6 per subject due to missed visits etc.) Measure-ment of plasma cytokines and chemokines with the Luminex 8-panel assay.	>IL-2, IL-4 IL-6, IL-8, IL-10, TNF-α, Interferon-γ(IFN-γ) and granulo-cyte-macrophage colony-stimulating factor (GM-CSF).	Mann-Whitney U test for comparisons of baseline values by relapse status. Wilcoxin signed rank test (within-subjects, paired samples design) to compare biomarkers at the visit preceding relapse and the visit after relapse. For those markers which differed significantly, logistic regression was used to test whether their baseline values predicted relapse, with age, sex, race, BMI, smoking and medication as covariates.	>Prospe-ctive design to assess group differences in cytokines levels of patients who relapsed and those who did not, as well as longitudi-nal variation of cytokine levels within the relapse group.	Mann-Whitney U tests revealed no significant differences based on relapse status (IL-2: p = 0.95, IL-4: p = 0.95, IL-6: p = 0.6, IL-8: p = 0.4, IL-10: p = 0.555, TNF-α: p = 0.787, IFN-γ: p = 0.07(trend that did not reach significance, GM-CSF: p = 0.97). Paired data for individual biomarkers (pre and post relapse, 4.4 weeks average temporal distance) revealed a significant decrease in IL-6(p=0.019) and IFN-γ (p=0.012). Logistic regression showed that neither of those at baseline was a predictor of relapse. (IL-6: OR=1.007, 95% CI 0.997–1.017, p=0.157, IFN- γ: OR=1.004, 95% CI 0.996–1.012, p=0.294)	No group differences between relapsed and non-relapsed patients were reported. Within the relapse group, there were significant decreases in IL-6 and INF-γ levels, when comparing pre and post relapse values, implicating both as potential state markers for relapse. However, baseline values of both those cytokines did not predict relapse.
Lin et al., 2023 (38) (China)	Cross sectional	n = 64 SCZ patients (34 FEP, 30 recurrent)	Blood samples and identification of metabolites with the Automatic Statistical Identification in Complex Spectra package. The relationship between traits and metabolites was examined with Weighted correlation network analysis.	>LC-MS (Liquid Chromato-graphy-Mass Spectro-metry) and HNMR (Hydrogen-Nuclear Magnetic resonance) metabolo-mics: modules or “networks” of different metabo-lites	WGCNA (Weighted correlation network analysis), MCODE (Molecular Complex Detection), CytoHubba and Lilikoi algorithms were used to identify weighted co-expression networks that were then correlated with traits. Student’s t test (for differences between two groups) and one-way ANOVA (among three groups), with p 0.05 as the significance threshold.	>Using weighted correlation networks to identify clusters of metaboli-tes associated with SCZ relapse.	WGCNA using a total of 458 metabolites, yielded 4 different modules. The turquoise module, containing 317 metabolites, resulted in the highest module-trait correlation (Pearson r = -0.78, p <0.0001. Trait refers to the status of the subjects, i.e. FEP, recurrent patients. Phenylalanylphenylalanine isolated as the key biomarker. One-way ANOVA revealed significantly lower phenylalanylphenylalanine levels in recurrent compared to FEP patients (p <0.05).	Weighted correlation network analysis revealed a cluster of 317 metabolites which correlated with clinical condition (FEP, recurrent patients and healthy controls). Phenylalanylphenylalanine was isolated as a potential state biomarker, as it differed between FEP and recurrent patients.
Isayeva et al., 2024 (39) (Italy)	Prospective (4 samples, every 6 months for 2 years)	n = 105 patients with 64 SCZ and 41 Schizoaffe-ctive Disorder, according to DSM-IV	Blood sample every 6 months for 2 years and BDNF assessed with ELISA. Clinical evaluation every visit via PANNS and PSP for social functioning.	>serum Brain-Derived Neurotro-phic Factor (BDNF)	Linear mixed effects model, with clinical remission, functional remission and relapse at various timepoints (T1 = 6 months etc.) as the independent variable (age, sex as covariates) and serum BDNF as the dependent variable	>Correlating baseline BDNF and BDNF variation in time with the state of maintained remission or relapse.	There was no statistically significant association between Clinical remission at 6,12,18 or 24 months, or recovery at 6 and 12 months with serum BDNF levels.(Z1 = 0.623, p1 = 0.533/Z2 = − 0.526, p2 = 0.599/Z3 = 0.073, p3 = 0.942/Z4 = 0.399, p4 = 0.689/Z5 = 0.474, p5 = 0.635/Z6 = − 0.521, p6 = 0.603) However, there was a significant correlation between baseline BDNF levels for subjects in clinical remission at 6 months (z = − 2.543, p = 0.011).	Longitudinal variation in serum BDNF did not correlate with the level of clinical or functional remission. Baseline BDNF levels significantly correlated with maintained remission for at least 6 months.

**Table 6:** Main points regarding sample, methodology, analysis, and results for papers examining neuroimaging/neurophysiology biomarkers.

Study/Country	Study Design	Sample (Diagnosis)	Data Collection Process	Biomar-kers examined	Analysis Tools	Main objectives	Statistical Results	Synthesis of main findings	
Lieberman et al., 2001 (40) (USA)	Prospective	n = 107 patients at baseline (SCZ or SAF) 51 patients had MRI data after at least 12 months of follow-up (study attrition)	structural MRI scans at baseline and every 18 months during follow-up Clinical follow-up for up to 6 years via SADS, SANS, CGI, and EPS scales.	structural MRI, various regional volumes (sub-cortical structures not included)	mixed model MANCOVA (multivariate analysis of covariance), encompassing effects at baseline, effects at the last MRI scan, as well as the effect of change between the two time points. The following characteristics was utilized: volume of each region was the dependent variable, while gender and group (poor vs good outcome, patients vs controls) were the between-subject factors. Age, interscan interval, sex and height were covariates.	Assessing longitudi-nal variation in MRI cortical volumes and their correlation with illness course (good versus poor outcome, defined by symptom recurrence)	Clinical assessments classified 64% of patients in the good outcome group and 36% in the poor outcome group. MANCOVA identified the following effects: There was no significant difference in baseline total ventricular volume between poor outcome and good outcome patients (F = 0.01, p = 0.92). There was a significant group-by-time interaction (F = 5.10, p = 0.0089). The ventricles of poor outcome patients increased in volume, something that did not occur in the good outcome group. *Post-hoc* comparisons of change scores in ventricular volume between poor and good outcome groups revealed a significant difference (F = 9.69, p = 0.0028).	Ventricular enlargement was significant in patients who did not respond to treatment, or relapsed during follow-up, compared to patients who achieved maintained remission. These structural changes in time could be associated with the presence and persistence of SCZ symptoms.	
de Castro-Manglano et al., 2011 (41) (Spain)	Prospective (regarding clinical follow up, however, only 1 MRI scan was performed)	n = 28 patients (SCZ and affective disorders, ICD-10 F20 and F30-39)	Baseline structural MRI scan. Each voxel represented the average amount of local grey (GM) or white matter. Clinical assessment was performed at baseline and after 3 years, via PANNS, HDRS, CGI and other scales.	>structural MRI, grey matter (GM) volume	two-sample t-test at baseline between patients with poor outcome versus good outcome (no apparent remission of symptoms, continuous hospitalization, unemployment)	>Compar-ing GM values between patients with good versus poor outcome.	two-sample t-test showed that the GM volume, specifically in the right hippocampus, was lower in the group of patients with poor outcome. (Z = 4.42, p < 0.001, uncorrected)	Lower Grey Matter volume in the right hippocampus was correlated with poorer outcome and thus could be associated with acute relapse in chronic patients.	
Yamadar et al., 2013 (42) (USA)	Cross sectional	n = 86 patients (DSM-IV-TR Axis I Disorders), divided into three groups: 13 FEP, 27 relapsed, 46 chronic stable	SORT (Semantic association retrieval task), where a pair of words is presented, that either elicits a third word (retrieval trial) or does not (non-retrieval trials). An example is the word pair “honey and “stings” which triggers the word “bees”. For the sort task, subjects completed a total of 92 trials (46 retrieval, 46 non-retrieval), while getting the fMRI scan.	>Accuracy (correct classifica-tion of retrieval or not) and reaction time in the SORT behavio-ral task. fMRI scan	Accuracy, defined as the correct number of responses for retrieval over the total number of retrieval trials, summed with the correct number of responses for non-retrieval over the total number of non-retrieval trials, was analyzed with a 2-condition (Retrieval, No-Retrieval) x 4-group mixed ANOVA. Reaction time (RT) was analyzed with a 2-accuracy (hit, miss) x 2-condition (Retrieval, No-Retrieval) x 3-group mixed ANOVA. Group differences were further evaluated (pairwise *post-hoc* comparisons). Contrast values in fMRI were obtained by creating spherical regions of interest around peak activity.	>Assessing differe-nces in a behavioral paradigm between FEP, chronic stable, and relapsed patients and simulta-neous alterations in inferior parietal lobule (IPL) activation in fMRI.	Mixed ANOVA for accuracy showed no differences between any of the SCZ groups (all p > 0.774). Mixed ANOVA for RT showed significantly faster RT ins the FEP and chronic stable groups when compared to the relapse group. (p < 0.01 and p= 0.016) Regarding fMRI data, left and right IPL activity was not significantly different between any of the SCZ groups. (left IPL: all p > 0.304, right IPL: all p > 0.699)	While fMRI activity in the left and right inferior parietal lobules has been shown to correlate with symptom severity (PANNS scores), it did not present any characteristic differences in relapsed patients, compared to first episode, or chronic stable patients. Regarding behavioral data, while accuracy did not correlate with group, RTs were significantly longer in the relapse group compared to both other groups, indicating that RT could be a useful metric in relapse identification.	
Nieuwenhuis et al., 2016 (43) (Nether-lands, UK, Brazil, Australia, Spain)	Prospective (regarding clinical follow up, however, only 1 MRI scan was performed)	n = 389 patients from multiple centers (SCZ spectrum). Of those, only those with a continuous or remitting illness course were included in the study (n = 212).	baseline structural MRI scan, calculation of spatially normalized grey matter probabilities Clinical follow-up for 3-7 years (multi-center study)	>structural MRI, grey matter (GM) probabili-ty in each of 170000 voxels per subject	A support vector machine (SVM) classifier was used to distinguish between patients with a continuous and patients with a remitting course of illness. Classification accuracy was evaluated via calculation of the positive and negative predictive accuracy.	>Classify-ing individuals into groups of different illness courses using machine learning (SVM) on baseline MRI scan data.	When including data from all centers, the classification accuracy did not differ from chance level. (52% positive and 52% negative predictive accuracy). When examining data from each center individually, the classification was significantly more accurate than chance level (68% PPA, 70% NPA, p < 0.02, p< 0.007) in only one center (London, UK).	Classification of patients into groups of different illness course based on grey matter density was not more accurate than chance level in the multi-center analysis, while in the single-center analysis, classification accuracy reached significance in only one center.	
Kim et al., 2020 (44) (Korea)	Prospective (2 PET scans, before and after discontinua-tion of medication)	n = 25 patients (DSM-IV-TR Axis I Disorders, FEP)	[18F]DOPA PET scans at baseline and after 6 weeks (i.e. two weeks after the 4 week medication discontinua-tion process). A [11C]raclo-pride PET scan was performed a week after the second [18F]DOPA PET scan. Clinical follow-up for up to 12 weeks, via PANNS, BPRS.	>[18F]DOPA PET and A [11C]raclopride PET scans, influx rate constants (Ki[cer] (l/min)) relative to the cerebel-lum, tracer binding potential (BP[ND]) to dopamine D2/3 receptors in the striatum	Linear mixed effects model to test the effect of group on Ki[cer] and BP[ND]. Group, modeled as a categorical variable: 1 =healthy controls, 2 = patients without relapse, 3 = patients with relapse) and the time point of PET imaging were modeled as fixed effects, and subjects were modeled as random effects. Pearson correlation between baseline Ki[cer] and the time to relapse.	>Examining longitudi-nal changes in Ki[cer] and BP[ND] values obtained from PET scans and their differe-nces between patients who relapsed versus those who did not.	Linear mixed effects models yielded a significant group by time effect for changes in Ki[cer] values between the three groups. (Week*Group: F = 4.827, df = 2,253.193, p = 0.009). Ki[cer] values were not significantly different among groups at baseline (F = 0.467, df = 2,211.080, p = 0.628), but were at week 6 (F = 3.512, df = 2,202.165, p = 0.032) *Post-hoc* pairwise comparisons revealed significant differences between Relapse and Non-relapse (p = 0.043) and between healthy controls and Non-relapse (p = 0.019), but not between Relapse and healthy controls (p = 0.854). In the Relapse group, a significant negative correlation was observed between baseline Ki[cer] values and time elapsed until relapse (R squared = 0.518, p = 0.018). No differences were observed in BP[ND] among the three groups (F = 1.402, df =2,32.000, p = 0.261).	Changes in striatal dopamine levels were found to correlate with psychotic relapse, and the time elapsed until it occurred. These results point at aberrant dopamine autoregulation as a contributing factor for psychotic relapse.
Mi et al., 2021 (45) (China)	Cross sectional	n = 32 patients (SCZ, DSM-IV or V)	EEG acquisition, while subjects performed a double oddball paradigm, with da/as the frequent stimulus and ba/and du/as the deviant ones, eliciting the consonant and vowel Mismatch Negativity respectively. Clinical outcomes were assessed retrospecti-vely based on previous interviews (PANNS, GAF).	>EEG, vowel and conso-nant induced phonetic Mismatch Negativity (MMN)	Pearson correlation between phonetic MMN reduction in 9 channels (F3, Fz, F4, FC3, FCz, FC4, C3, Cz, C4) and illness relapse.	>Correla-ting phonetic MMN amplitude with illness relapse (hospitali-zations, medica-tion dosage increase of 25% or more, suicidal ideation)	For the consonant induced MMN (da-ba) there was no significant correlation in any of the 9 electrodes with illness relapse (all p > 0.18). For the vowel induced MMN (da-du) significant differences were found in 6 of the 9 electrodes. F3: r = 0.428, p = 0.015//Fz: r = 0.420, p = 0.017//FC3: r = 0.435, p = 0.013//FCz: r = 0.380, p = 0.032//FC4: r = 0.370, p = 0.037//C3: r = 0.367, p = 0.039.	Significant correlations between vowel phonetic MMN amplitude and illness relapse, implicate automatic speech processing deficits as a potential contributor in relapse occurrence.
Solanes et al., 2022 (46) (Spain)	Prospective (regarding clinical follow up, however, only 1 MRI scan was performed)	n = 277 patients (SCZ, SAF, Bipolar Disorder and other)/120 HC	Structural MRI at baseline (T1-weighted gradient-echo sequence). Clinical assessment for up to 24 months, or until relapse, defined via PANNS.	>structural MRI image	Data was segmented into a train set, to obtain model parameters and a test set, for which relapse was predicted. Lasso regression was utilized, and each parameter value was multiplied by the corresponding variable value for each individual. After obtaining the sum of these terms, if the result was <= 0, the individual was classified as low risk for relapse, and if it was >0, as high risk. To test classification accuracy, a mixed-effects Cox proportional hazards regression model was used.	>Using machine learning to classify individuals into two groups, correspo-nding to low and high risk of relapse, based on baseline MRI images.	16 relapses were recorded (9.4% relapse rate in 24 months), corresponding to 70% statistical power to detect a Hazard Ratio of 4.3. Lasso regression selected the following key variables: diagnosis of schizoaffective disorder, lack of difficulty in abstract thinking and poor impulse control, and the increase or decrease of unmodulated and modulated gray and white matter in several brain regions. Cox regression resulted in a Hazard Ratio of 4.58, meaning that patients in the high risk group had almost 5 times more risk to relapse. This result reached statistical significance. (Hazard Ratio 95% confidence interval = 1.01–20.74, Z = 1.98, p = 0.048)	While a model using combined clinical and MRI parameters did classify low vs high risk for relapse, that was not possible with the sole use of MRI data. Furthermore, issues of statistical power arose in the study, given the low number of relapse events recorded. To sum up, evidence from this study suggests that MRI could combine with clinical follow-up, resulting in more precise risk stratification for psychotic relapse.
Sasabaya-shi et al., 2022 (47) (Japan)	Prospective (regarding clinical follow up, however, only 1 MRI scan was performed)	n = 52 patients (SCZ, according to ICD-10)/19 relapsed - 33 did not relapse	Structural MRI at baseline. Calculation of the local gyrification index (LGI), which refers to the cortical “folding”, or the amount buried within troughs of the cortex, in 800 regions of interest. Clinical assessment for up to 3 years, or until relapse.	>structural MRI image, Local gyrifica-tion index (LGI)	Group differences were assessed via one-way ANOVA or the chi-squared test. Monte Carlo simulations were run to perform multiple comparisons (10000 iterations for each comparison). p < 0.05 defined significant clusters.	>Compar-ing LGI values across 800 regions, between the group of patients who relapsed and those who did not.	The relapse group exhibited a significantly higher LGI in 3 clusters with sizes (in mm^2) of 4022.36, 707.22, 646.85. The first cluster (p = 0.0001) corresponded to the left precuneus and cuneus cortex, isthmus cingulate gyrus, pericalcarine cortex, and lingual gyrus. The second cluster (p = 0.0161) corresponded to the left superior parietal lobule. The third cluster (p = 0.0328) corresponded to the right precuneus cortex, posterior and isthmus cingulate gyrus.	Significant differences in LGI between the relapse and non-relapse groups across various regions, could hint at neurodevelopmental anomalies in the pathogenesis of psychotic relapse.
Rubio et al., 2022 (48) (USA)	Cross sectional	n = 50 patients (SCZ, SAF, Bipolar Disorder I and others, according to DSM-IV. Patients were split into the Break-through Psychosis group (BAMM: breakthrough on antipsychotic maintenance medication) consisting of 23 individuals and the Antipsychotic free group (APF, relapse after voluntary discontinuation of treatment), consisting of 27 individuals	Approxima-tely 20 minutes of resting state (awake, eyes closed) fMRI Calculation of the Striatal Connectivity Index (SCI), which involved measuring functional connectivity of subregions in the striatum, creating connectivity maps and extracting the strength in 91 of those connections to obtain the SCI. (single value per scan, or per participant)	>resting state fMRI, Striatal Connecti-vity Index (SCI)	Linear regression model, with group status (Breakthrough psychosis, psychotic relapse after discontinuation of medication, healthy controls) as the independent variable, SCI as the dependent variable and sex and age as covariates. *Post hoc* analysis (re-run of the same model), including only SCZ and SAF diagnosis.	>Examining differen-ces in the SCI between acutely relapsed patients and controls, as well as differences between patients who relapsed despite continuation of mainte-nance treatment and those who relapsed after disconti-nuation of treatment.	Group comparisons for the entire patient group yielded the following results: SCI values were significantly lower in the BAMM group than in the APF patient group as well as the HC group. (APF: Cohen’s d = 0.58, linear regression: ß = 0.86, p = 0.032, HC: d = 0.99, linear regression: ß = 1.47, p < 0.001). When comparing APF to HC there was a trend toward lower values in the APF group, that did not reach the p < 0.05 significance level. (Cohen’s d = 0.44, linear regression: ß =0.61, p = 0.09). *Post hoc* analysis including only SCZ and SAF patients resulted in the same significant difference between BAMM and HC (p < 0.001), only now both the trend differences between BAMM and APF, as well as APF and HC, did not reach significance. (p = 0.07 and p = 0.08 respectively)	This study provides evidence that striatal functional connectivity could be impaired (lower SCI value) in individuals experiencing psychotic relapse. Differences in SCI values were significantly more prominent for the group of patients who relapsed despite guaranteed continuation of antipsychotic treatment, which could hint at SCI as a marker of treatment non-response.
Odkhuu et al., 2023 (49) (Korea)	Prospective (regarding clinical follow up, however, only 1 fMRI scan was performed)	n = 30 patients (SCZ spectrum or other, DSM-IV, recovered at recruitment). Patients were split into two groups, those who relapsed and those who did not	5 minutes of resting state fMRI Calculation of functional connectivity (FC) matrices, to obtain the Global FC strength (mean of all pairwise correlation coefficients) Clinical follow-up every 2 months for 1-2 years, via PANNS, SOFAS and the Calgary Depression Scale for SCZ.	>resting state fMRI, Global Functio-nal Connecti-vity strength (GFC)	one-way ANOVA for group differences in GFC between patients and healthy controls (first comparison) and relapsed patients, relapse free patients and healthy controls (second comparison).	>Compa-ring GFC values between chronic patients and controls, as well as within the patient group, between individuals who relapsed and those who did not.	Regarding patient- HC differences in GFC, the ANOVA showed that GFC was significantly higher in patients (patients: 0.240 ± 0.035, HC: 0.223 ± 0.028, p < 0.0001). ANOVA for relapsed and relapse free patients showed a significantly higher GFC in relapsed patients (Relapse: 0.290 ± 0.040, Non-relapse: 0.216 ± 0.039, p = 0.0002). For relapsed patients and controls the same significant difference was observed. (Relapse: 0.290 ± 0.040, HC: 0.223 ± 0.028, p < 0.0001). Comparison of the non-relapse group with HC did not yield significant differences.	The results of this study implicate global metrics in baseline resting state fMRI as state markers for relapse, since GFC values were found to differ significantly in the group of patients who subsequently relapsed, compared to both patients who achieved prolonged remission and healthy controls.

**Table 7:** Synthesized findings from studies using cognitive/behavioral markers as predictors of relapse.

Study/Country	Study Design	Sample (Diagnosis)	Data Collection Process	Biomar-kers examined	Analysis Tools	Main objectives	Statistical Results	Synthesis of main findings	
Chen et al.2005 (50) (Hong Kong)	Prospective (2 cognitive assess-ments, at admission and after stabilization, clinical follow-up for 3 years)	n = 93 patients (SCZ, SAF, Schizophreniform disorder according to DSM-IV)	Cognitive assess-ments at admission and after stabilization included the forward digit span test, the Logical Memory test, the Modified Wisconsin Card Sorting Test and a semantic fluency test. Clinical follow-up every 4 months for 3 years via PANNS.	>Various cognitive factors	A multiple binary logistic regression model was used with relapse (categorical, 0 or 1) as the dependent variable and standardized Z-scores for cognitive function scores as independent variables.	>Examining whether scores in various cognitive tests related to memory, sematic association and executive function could predict the occurrence of relapse in a 3-year follow-up period.	40% of patients relapsed within the full 3 year follow-up period. (21% and 33% for years 1 and 2) In the multiple logistic regression model, only the preservative error in the Wisconsin test yielded a significant odds ratio of 2.46 (p = 0.027).	While both visual and verbal memory, as well as semantic fluency were not significantly associated with relapse, executive dysfunction as assessed by the Modified Wisconsin Card Sorting Test could be a predictor of adverse outcomes in SCZ.	
Rund et al., 2007 (51) (USA)	Prospective	n= 207 at baseline, n = 111 for the second-year follow-up assessment (SCZ, SAF and others, DSM-IV)	8(yearly) neuropsy-chological tests: 1) California Verbal Learning Test 2)Backward Masking Test 3)Wisconsin Card Sorting Test 4)Controlled Oral Word Association Task 5) Trail Making Test, A + B 6) Digit Span Test 7) IQ, three subscales from WAIS 8) Continuous Performance Test, Identical Pairs Version, and clinical follow-up via PANNS.	>Five indices of cognitive performance (mean z-score of the tests for each) Working Memory (WM), Executive Function (EF), Verbal Learning (VL), Impulsivi-ty (Im) and Motor Speed (MS).	Bivariate correlations between baseline values for the 5 indices and number or duration of relapse were assessed with Spearman’s rho (p < 0.01 was the threshold for significance). One-way repeated measures ANOVA with Bonferroni correction was used to assess the variation of cognitive indices mean value with time (baseline, 1 year, 2 years).	>Examining whether baseline values or differen-ces in time, of various cognitive perfor-mance indices, are correlated with number or duration of relapse.	No relationships were found between any of the 5 cognitive indices and duration of relapse. Number of relapses were split into 2 groups (relapses within the first and second year). Number of relapses in the second year did not correlate with any of the 5 cognitive indices. There were significant correlations for WM and VL with number of relapses in the first year. These correlations were significant for WM and VL values at baseline (r = -0.21, r = -0.2, both p < 0.01) and at year 1 (r = −0.27, r = −0.34, both p < 0.01).	While performance in none of the five cognitive domains correlated with number of relapses in the second year of the study, Working memory and Verbal learning performance at baseline and after one year did correlate with number of relapses within the first year. This could imply that premorbid cognitive level, or level during early stages of illness, could be a predictor of relapse.	
Birnbaum et al., 2019 (52) (USA)	Retrospe-ctive (longitudinal)	n = 51 patients (SCZ or SAF)	Patients were asked to download the entirety of their uploading history of the Facebook platform including messages, posts, photos, comments, shares and likes. A total of 52,815 Facebook posts was available for analysis.	>Linguistic (words associa-ted with negative emotions or death, words related to work or peers) changes apparent in Facebook posts	Wilcoxon ranked sign test to compare the usage frequency of specific words indicative of anger, swear or death, between periods of relapse and relative health. Support vector machines (SVMs) were used as predictive models, to classify an unspecified period of time into relapse or relative health, after training. For training, periods of 1,2 and 3 months were used. To augment the 1-month model, an ensemble model was also trained, again using 1-month intervals as training data.	>Testing whether changes in Facebook posting habits, as well as word choices in posts, can predict psychotic relapse.	Wilcoxon signed rank tests yielded significantly higher usage of words associated with death (p <0.0001), swear (p < 0.0001), negative affect (p < 0.001), hear (p <0.0001), and feel (p < 0.01). SVMs models predicted relapse with the following sensitivity(SE), specificity(SP), Positive predictive value(PPV) and negative predictive value(NPV). 1-month model: SE = 0.47, SP = 0.65, PPV = 0.66, NPV = 0.46. 2-month model: SE = 0.57, SP = 0.28, PPV = 0.41, NPV = 0.44. 3-month model: SE = 0.90, SP = 0.04, PPV = 0.37, NPV = 0.4. Ensemble model: SE = 0.38, SP = 0.71, PPV = 0.66, NPV = 0.44.	Linguistic changes were apparent between relapse and relative health periods, in the usage frequency of words related to negative emotions and death. However, none of the SVM predicted models achieved more than 0.66 positive predictive value, which could be explained by the fact that symptoms fluctuate on much smaller time scales than the 1-month minimum used to train the models.	
Birnbaum et al., 2020 (53) (USA)	Retrospe-ctive (longitudinal)	n = 42 (SCZ or SAF, DSM-IV)	Patients and controls retrieved their Google activity through a service called “takeout”. 32,733 search queries were analyzed. Data was segmented into 1-month periods and labeled as relapse or non-relapse.	>Freque-ncy and content of internet search activity.	Three different classifiers were used for relapse prediction, with 123 features (e.g. 1h-bin histograms, average search query length) for each. These were: random forest (RF), support vector machine (SVM) and gradient boosting (GB). AUC and mean accuracy were calculated to evaluate model performance.	>Testing whether changes in internet search activity patterns can predict psychotic relapse.	Regarding the relapse classifier, the mean accuracies and AUCs for the three types of classifier were: acc =0.63, AUC = 0.71 for SVM, acc = 0.61, AUC = 0.69 for RF and acc = 0.65, AUC = 0.71 for GB. Most significant features for the relapse classifier were the use of search words from the “sexual,” “health,” “hear,” “anger,” “sadness,” and “perception” categories, reductions in search length and search frequency prior to a relapse hospitalization.	The feasibility of using supervised machine-learning algorithms based on linguistic and other features in online search activity was explored in this study. The max AUC of 0.71, resulting from the Gradient Boost model, shows some, but not strong predictive power for relapse.
Tao et al.2023 (54) (Hong Kong)	Prospective	n = 110 patients (SCZ or non-affective psychosis diagnosis according to DSM-IV)	Monthly interviews over the phone for 1 year, which included working memory assessment (Visual Patterns Test, Letter-Number Sequencing test admini-stered via smartphone app) and potential relapse identifica-tion.	>Visual and verbal working memory	Two stage analysis, for a binary outcome of relapse or no relapse. In the first stage, a binary logistic regression model was run with each individual predictor as the independent variable. In the second stage, significant predictors from stage 1 were incorporated in a multiple logistic regression model, alongside clinical variables such as treatment adherence and resilience.	>Examining whether working memory deficits characterize or even precede relapse	Of the 17 cognitive markers examined at baseline, and the 4 differences (i.e. value at baseline - value at visit x), only one, the deterioration of performance in the Letter-Number Sequence (LNS) paradigm was significantly associated with relapse risk. (OR = 7.015, p = 0.004). This finding was corroborated in the multiple logistic regression model. (LNS deterioration OR = 9.445, p = 0.029).	Working memory deterioration two months prior to relapse, measured in the LNS task, was significantly associated with higher relapse risk. Baseline cognitive performance, as well as performance deterioration in the Visual Patterns Test did not predict relapse.

**Table 8:** Presentation of samples, data collection process, analysis tools and key findings from studies using passively collected data from wearable devices and smartphones.

Study/Country	Study Design	Sample (Diagnosis)	Data Collection Process	Biomar-kers examined	Analysis Tools	Main objectives	Statistical Results	Synthesis of main findings	
Ben-Zeev et al., 2017 (55)(USA)	Prospective	n = 5 patients, data collection was still ongoing (SCZ spectrum disorders)	Cross check data set: Samsing Galaxy S5 phones were provided to patients for 1 year, with the Cross check app recorded accelero-meter, GPS location, speech frequency and duration and number of calls. The app actively collects clinical data (question-naires)	>Accelerometer, speech frequency and duration, GPS location, number of phone calls, time spent on apps and phone unlocks.	Feasibility test-study. Cases selected for demonstrative purposes. No systematic analysis performed.	>Presenting a longitu-dinal patient monitoring protocol via smart-phone and assessing the feasibility of passively collected data from the phone to identify psychotic relapse.	No statistical tests were performed.	The feasibility of long-term patient monitoring via passively collected data from a smartphone is assessed in this study. Each of the 5 patients examined exhibited an abnormality in at least one of the continuously measured parameters, right before relapse. (e.g. patient 2 had a total behavioral shift in GPS data, where they stopped spending time at the identified primary location, most likely their home)	
Barnett et al., 2018 (56) (USA)	Prospective	n = 15 patients (SCZ, 5 experienced relapse)	Smart-phones were provided for 3 months (Beiwe app installed). Actively collected data from app prompts included psychotic symptom and mood self-assessment, while passively collected data included GPS location, accelerometer, calls and messages.	>Daily features extracted from accelerometer data, texts and calls, screen activity and GPS location ( 15 mobility and 16 sociability features).	Multivariate timeseries anomaly detection method, which included establishing a trend, calculating the error, or deviation from the trend, via Hotelling’s T squared test and finally a non-parametric transformation of the errors into Z scores by sorting them.	>Using anomaly detection on mobility and sociability features from passively collected smartphone data to identify relapse.	Average monthly anomaly rate for all patients was 1.8 for mobility features and 1.7 for sociability features. (authors report large between-subject variability) Only three of the five individuals who relapsed had sufficient data for analysis and in those, anomalies were increased by 71% compared to non-relapsed patients.	Preliminary results provide evidence for smartphone monitoring as an alternative, or at least a complementary method to clinical follow-up, to achieve early relapse detection.
Adler et al., 2020 (57) (USA)	Prospective	n = 60 patients (SCZ and Schizophrenia spectrum disorders, 18 relapse-42 non-relapse)	Cross check data set. (described above)	>Hourly features extracted from the complete dataset, including acceleration, location, sleep, app use, texts, calls, conversations and screen activity.	Various encoder-decoder neural network models trained to predict relapse based on anomaly detection in the pre-relapse (30 days) period. Training and test (used to evaluate model accuracy) data consisted of subsequences (length 1 day) which were classified as near relapse (within the preceding 30 days of a relapse episode) or relative health sequences. Sensitivity (or TPR), specificity and the ratio of True positive rate/False negative rate (TPR/FPR) was used for evaluation of model performance.	>Predicting relapse by anomaly detection in passively collected data from a smartphone, using unsupervised machine learning (autoencoders).	For the FNN AD(Fully Connected Neural Network Autoencoder) model type the highest performing model achieved a median(Inter Quartile Range) sensitivity of 0.25 (0.15 - 1.00) and specificity of 0.88 (0.14 - 0.96) in the cross-validation procedure. (Monte-Carlo simulations, 100 iterations, with different training data) For the GRU Seq2Seq (gated recurrent unit sequence-to-sequence) model type, the highest performing model achieved a sensitivity of 0.29 (0.08-0.83) and a specificity of 0.86 (0.24-0.90).	Encoder-decoder neural networks predict relapse based on anomalies up to 1-month prior to relapse with high specificity but low sensitivity. High specificity indicates that behavioral changes do occur in the pre-relapse periods, while low sensitivity could be explained by short term variation of the examined markers. (i.e. changes occur only during certain, but not all, days in the month preceding relapse)	
Henson et al., 2021 (58) (USA)	Prospective	n = 83 patients (SCZ). 63 patients provided more than 2 weeks of data and were included in analyses.	Smart-phones were provided for 3-12 months, with the Beiwe and mindLAMP apps installed. Same data as in [], plus sleep duration and cognitive features (Jewels Beta A and B) were analyzed. Clinical assess-ments every 1-3 months via PHQ-9, GAD-7, PANSS and CGI.	>Daily features extracted from passively collected smartphone data, including 16 sociability features, 15 mobility features, sleep duration, 2 cognitive features and 6 survey features.	Multivariate timeseries anomaly detection Social distancing protocols due to COVID-19 were a potential confounding factor.	>Using anomaly detection on mobility and sociability features from passively collected smartphone data to identify relapse.	1006 anomalies recorded in 73 participants, with a per subject anomaly rate ranging from 0 to 4.7. Paired anomalies (i.e. simultaneous anomalies in more than one feature) occurred in 28 patients and 11 HC. Paired anomalies exhibited a sensitivity of 89%, a specificity of 75%, a positive predictive value of 60%, and a negative predictive value of 94%, in distinguishing relapse from non-relapse.	Further evidence is provided in favor of detecting relapse via remote monitoring, by tracking anomalies in mobility, sociability, cognition and sleep via smartphone.
Zhou et al.2022 (59) (USA)	Prospective	n = 63 patients (SCZ and Schizophrenia spectrum disorders, 27 total relapses, multiple instances for some patients)	Cross check data set.	>Hourly features extracted from the complete dataset, including acceleration, location, sleep, app use, texts, calls, conversations and screen activity.	Anomaly detection with two clustering methods, Gaussian mixture model (GMM) and partition around medoids (PAM). For each point (one day of data), a weighted average likelihood score was calculated, indicative of the proximity of each point, to populous clusters, i.e. its regularity. A low score indicates an anomaly. (4 weeks predict relapse on the 5-th, F2 scores for model predictive value)	>Predicting relapse by anomaly detection via clustering analysis.	The best clustering model (based on cluster overlap and other metrics) was a 9-cluster model, including clusters with a size (in days of data) ranging from 412 to 5217. The largest cluster corresponded to no app use, high conversation and text messaging and average values for other features. The highest F2 score of 0.23, which was obtained when combining cluster data with baseline clinical data, was significantly higher than the F2 score of random classification (0.042).	Behavioral signatures can be extracted for passively collected smartphone data via the use of clustering algorithms. Alterations in these signatures, or anomalies, hold some predictive value for oncoming psychotic relapse.	
Zlatintsi et al., 2022 (60) (Greece)	Prospective	n= 24 patients, study was still ongoing (SCZ, SAF, BP1 according to DSM-V)	Samsung Gear S3 Frontier smart-watches were provided for up to two years, continu-ously recording heart rate (RR intervals), accelero-meter, gyroscope, steps and sleep data. Monthly clinical evaluations via PANNS, GAF, IPAQ, WHODAS II and cognitive tests such as the Go-No-Go paradigm.	>5-min intervals of heart rate, accelero-meter, and gyrosco-pe data, tagged as sleep or non-sleep and as relapse, pre-relapse (1-month prior) and relative health.	4 different autoencoder architectures were used: Transformers, Fully connected Neural Networks (FNN), Convolution Neural Networks (CNN) and Gated Recurrent Units (GRU). 1569 days of data (in 5-min intervals) from 10 patients were used in analyses. Data from relative health periods was split (60%-20%-20%) into training, validation, and test data. No relapse data was used for training, but only for testing. Receiver Operating Characteristic Area Under Curve (ROC AUC) and Precision-Recall Area Under Curve (PR AUC) were used to evaluate model performance.	>Using unsuper-vised machine learning models to predict relapse on a per person and on a general basis, with input data from smart-watch sensors, including heart rate, accelero-meter, and gyroscope measure-ments.	In the personalized analysis the median (across 10 subjects) ROC AUC values were 0.57 for the FNN architecture, 0.61 for CNN, 0.47 for Transformer and 0.54 for GRU. 0.50 was the ROC AUC value of random classification. PR AUC median values were 0.75 for FNN, 0.76 for CNN, 0.61 for Transformers and 0.71 for GRU. 0.68 was the PR AUC for random classification. For both metrics, the CNN architecture had the better performance. Regarding the global analysis, where data from all patients was pooled together for model training, the ROC AUC was 0.77 for FNN, 0.71 for CNN, 0.76 for Transformers and 0.73 for GRU. (random classification: 0.68) PR AUC was 0.62 for FNN, 0.58 for CNN, 0.52 for Transformers and 0.57 for GRU. (random classification; 0.50). In the global analysis an FNN model was the best performing one.	Preliminary results from the e-Prevention study show evidence that supports personalized relapse detection and prediction via heart rate, accelerometer, and gyroscope data. Data collection with smartwatches is non-invasive and does not pose a significant hindrance in patient everyday routines, which ensures scalability of this approach.
Lamichla-ne et al., 2023 (61) (USA)	Prospective	n = 63 patients (SCZ and Schizophrenia spectrum disorders, 27 total relapses, multiple instances for some patients)	Cross check data set.	>Hourly features extracted from the complete dataset, including acceleration, location, sleep, app use, texts, calls, conversations and screen activity.	Relapse (binary classification) at the 5-th week was predicted based on data from the preceding 4 weeks. Naive bayes was used for classification, with Balanced Random Forests and EasyEnsemble as alternatives, for comparison. F2 scores were calculated to assess model predictive value.	>Predicting relapse with supervised machine learning classifiers such as naive bayes, balanced random forests and EasyEnseble classifiers.	Naive bayes achieved the highest F2 score = 0.083, followed by Balanced Random Forests with F2 = 0.042 and EasyEnsemble with F2 = 0.034. A random classification resulted in F2 = 0.02 +/- 0.024.	A naive bayes classifier was used for classification of data as relapse or non-relapse, based on features extracted from passively collected data from a smartphone. F2 scores indicate a small, but statistically different than chance level predictive power for relapse.
Cohen et al., 2023 (62) (USA, India)	Prospective	n = 76 patients (SCZ according to DSM-V, 20 relapsed- 56 did not)/56 HC	Smartphones were provided for up to 12 months, with mindLAMP app installed. Accelero-meter, GPS, sleep, and screen activity data collected.	>Daily sociability, mobility, and sleep features.	Multivariate timeseries anomaly detection p value calculation for each day, with p < 0.005 considered as an anomaly. p values calculated with the same method as in (59). Anomaly detection as a prediction method was compared to a naive logistic regression model.	>Using anomaly detection to detect and predict psychotic relapse.	20 relapses and 188 significant anomalies (p < 0.005) were detected. Anomaly rate was higher (2.12 times) in the month preceding relapse, compared to the 1.5 times higher ratios predicted by the logistic regression model. Of the detected anomalies, 13(6.9%) were associated with relapse (true positives). Sensitivity and specificity for the anomaly detection model was 0.006 and 0.997.	The high specificity of anomaly detection models, indicates that relapses are characterized by behavioral changes, the low sensitivity of these models requires the addition of more biometric or clinical data.

It should be noted that in many studies relapse prediction constitutes only one of the pursued goals. Here we focus solely on this axis, disregarding unrelated findings, for example any group differences between patients and healthy individuals, which relate to diagnosis and not relapse.

### 3.1 Genetic biomarker subgroup

In **Table 4** (below) synthesized findings from 4 (9%) studies using genetic biomarkers are depicted.

Meier et al. (21) analyzed approximately 3000 genomic risk profiles (GPRS), which is calculated using alleles at thousands of different loci. The study examined correlations between the GPRS and admission frequency, specifically for SCZ relapse. The authors reported that for a high number of single-nucleotide polymorphisms included in the GPRS, it correlated significantly with number of admissions. Segura et al. (24) calculate the polygenic risk score (PRS) [metric similar to GPRS in (21)], but find no significant differences in the PRS between SCZ patients who relapsed in a span of three years and those who did not. Pawelczyk et al. (22) calculated the length of telomeres (regions at the end of chromosomes), whose faster than normal decay has been linked to a plethora of non-psychiatric disorders. Their results indicate that shorter telomere length correlates with a higher number of psychotic episodes. Gasso et al. (23) implemented a prospective design and collected the total RNA (337.000 transcripts and variants corresponding to 20.800 genes) twice, at baseline and after 3 years or at relapse if it occurred, in 91 patients with SCZ. They created gene connectivity networks or clusters, consisting of 41 to 5627 different genes. This approach captures co-expression of certain genes which form larger coalitions and via implementation of preservation analysis, the stability of these networks in time can be assessed. The authors identified two main networks that were semi-conserved after 3 years, but their ability to predict relapse did not reach significance. Patients were then split based on gene expression of the most stable network, with those at the highest percentiles showing higher relapse risk.

### 3.2 Blood-based biomarker subgroup

In this next category we review the 15 (36%) studies using non-genetic biomarkers derived from blood samples. **Table 5** showcases aggregated findings from our analysis.

In (30, 34, 39) prospective designs (2-3 years, 221, 69, and 105 SCZ patients respectively) are used to assess the relation of BDNF and NGF with psychotic relapse. In Pillay et al. (30), Martínez-Pinteño et al. (34) as well as Isayeva et al. (39), both BDNF and NGF do not predict relapse, neither in ROC curve analyses, nor in linear effects models. Borovcanin et al. (27), Luo et al. (33), and Miller et al. (37) assess the predictive value of various cytokines, such as IL-2, IL-4, IL-6, IL-8, IL-23 TNF-α and others (see **Table 5**). Borovcanin et al. (27) implemented a cross-sectional design to compare IL-23 levels between 78 FEP and 47 relapsed patients. No significant differences in IL-23 levels between FEP and relapsed patients were reported. Luo et al. (33) collected blood samples at admission and discharge from 68 patients and compared the levels of IL-6, IL-18, INF-γ, and TNF-α. Paired t-tests revealed significant differences only in IL-6 levels. Miller et al. (37) collected blood samples with a maximum frequency of once every 3 weeks for up to 30 months in 200 patients (70 relapses during the study). Group comparisons between relapsed and non-relapsed patients yielded no significant results. However, comparisons between pre- and post-relapse cytokine levels within the relapse groups revealed significant decreases in IL-6 and INF-γ values. Ozdin and Boke (32) retrospectively compared ratios involving white blood cells, platelets, neutrophils, monocytes, and lymphocytes in 105 patients, during admission and discharge from the hospital. Multiple markers indicative of inflammation, most notably white blood cells and the monocyte to lymphocyte ratio, were found to be significantly elevated during relapse compared to remission. Piotrowski et al. (31) use a cross-sectional design comparing FEP patients (42), with relapsed patients (25). They use the allostatic index (AI), which encompasses a variety of biomarkers, namely Systolic and Diastolic blood pressure, BMI, waste to hip ratio, high sensitivity CRP, fibrinogen, albumin, fasting glucose and insulin, total cholesterol, LDL, HDL, triglycerides, cortisol and DHEA-S. ANCOVA revealed significant differences between relapsed patients and FEP patients, with blood pressure and waste to hip ratio being the most important individual contributors. Marques and Ouakinin (35) prospectively followed a sample of 60 patients, measuring unconjugated bilirubin (UCB) during relapse and remission. UCB differed significantly (ANOVA) between relapse and remission. Fabrazzo et al. (36) performed a cross-sectional comparison between 74 acutely relapsed patients and 78 stable outpatients measuring Vitamin D and Parathyroid Hormone (PTH). Both Vitamin D and PTH were found to be significantly lower in patients experiencing relapse. Morera-Fumero et al. (28) collected blood samples from43 patients at admission and discharge and calculated the Total Antioxidant Capacity (TAC), which refers to the antioxidant capacity of water-soluble molecules such as albumin, caeruloplasmin and other proteins. TAC values did not differ significantly between relapse and remission. The final four studies, namely (25, 26, 29, 38) use biochemistry markers. Kaddurah-Daouk et al. (25) compared the plasma lipid profiles (5 phospholipids) of 20 patients experiencing relapse with 20 FEP patients. Wilcoxon rank sum tests revealed no significant differences between FEP and relapsed patients. Schwartz et al. (26) used multiplex immunoassays to obtain a panel of 191 proteins and small molecules in 77 patients, who were prospectively evaluated clinically (18 relapsed, 59 did not). Wilcoxon rank sum tests were applied to test for group differences, while random forest analysis was used for classification based on baseline biomarker values. Significant group differences were present in 27 molecules. Random forest analysis predicted the time to relapse (as a binary classification problem, i.e. short vs long term relapse) with 94.5% accuracy. Weight change alone predicted the same result at an accuracy of 83.4%. Szymona et al. (29) compared the levels of certain kynurenines (such as Kynurenic Acid and 3-Hydroxykynurenine), in blood samples of 51 patients between relapse and remission. Mann-Whitney U tests did not reveal significant differences within the patient group at the two time points. Lin et al. (38) used weighted correlation networks to identify clusters of metabolites (Liquid Chromatography-Mass Spectrometry metabolomics) that could differ between 34 first episode and 30 recurrent patients. Weighted correlation network analysis isolated a cluster of 317 metabolites correlating with status (FEP, recurrent patient), with phenylalanylphenylalanine being the single most influential predictor.

### 3.3 Neuroimaging/neurophysiology biomarker subgroup

We proceed with the findings of neuroimaging and neurophysiology studies, including structural Magnetic Resonance Imaging (MRI), functional MRI, electroencephalogram (EEG) and Positron Emission Tomography. Our search yielded 10 (24%) such papers, briefly summarized in **Table 6**.

In (40, 41, 43, 46, 47) metrics from baseline structural MRI scans are used to predict relapse. (Only in (40), repeat MRI scans were performed every 18 months). Liebermann et al. (40) performed structural MRI scans every 18 months on 107 patients (with 51 remaining in the study after a year). The first and last MRI scan of every individual was used in mixed model multivariate analysis of covariance, which showed significant ventricular enlargement in chronic patients when compared to those who achieved maintained remission. De Castro-Manglano et al. (41) used structural MRI scans to compute Grey Matter (GM) volume, which was then used for comparisons between patients with good versus poor outcome. Two sample t-tests showed that GM volume was significantly lower, most notably in the right hippocampus of patients experiencing poor outcomes. Nieuwenhuis et al. (43) performed a baseline MRI scan and clinically followed up on 212 patients for 3-7 years. They constructed a probability map for grey matter values in approximately 170.000 voxels and proceeded to apply a support vector machine classifier to test the feasibility of illness course classification based on baseline MRI data. Classification accuracy did not significantly differ from chance level when using aggregated data from 7 research centers. Solanes et al. (46) performed baseline MRI scans and clinically monitored 277 patients for up to one year aiming to develop a risk stratification framework based on MRI and clinical data. While only 16 relapse incidents were reported in the study, the authors report that Cox regression resulted in a 4.58 Hazard ratio for the high risk group, albeit in the combined clinical and biomarker model. The sole use of biomarker data did not result in accurate high versus low risk classification. Sasabayashi et al. (47) calculated the Local Gyrification Index (LGI) from the MRI scans of 52 patients, 19 of which experienced relapse during the 3 year follow up period. The LGI is a measure of cortical folding (i.e. area of peaks and troughs). Significant differences between relapsed and non-relapsed patients were reported in 3 of 800 regions of interest, providing some evidence that higher LGI, associated with neurodevelopmental anomalies, could play a role in relapse.

In (42, 48, 49), the authors utilize data from functional MRI scans. Yamadar et al. (42) implemented a cross-sectional design with 13 FEP and 27 acutely relapsed patients, who performed the Semantic Association Retrieval Task (SORT), while getting a functional MRI scan. The main region of interest was the inferior parietal lobule. Behavioral results were mixed (no differences in accuracy but significant faster reaction times for the FEP group compared to the relapse group), while fMRI results showed no differences in inferior parietal lobule activation levels. Rubio et al. (48) obtained 20 minute resting state fMRI scans of 50 acutely relapsed patients, further split into those who relapsed despite taking antipsychotic medication (23) and those not receiving treatment (27). After calculating the Striatal Connectivity Index (SCI), linear regression models were applied to test for differences between the two patient groups, as well as between all relapsed patients and healthy controls. SCI was significantly lower in patients, with the difference being more prominent in individuals experiencing relapse despite treatment. Odkhuu et al. (49) gathered 5 minutes of resting state fMRI in 30 patients, split into those who relapsed and those who did not. The Global Functional Connectivity Strength (GFC) was found to differ significantly between relapsed and non-relapsed patients via one-way ANOVA.

Kim et al. (44) gathered two PET scans from 25 patients, before and after medication discontinuation. Linear mixed effects models showed significant group by time differences in the influx rate constant related to the cerebellum (Ki[cer]), between patients who relapsed and those who did not. Within the relapse group, baseline Ki[cer] correlated negatively with time elapsed until relapse.

Mi et al. (45) collected EEG data from 32 patients during the vowel and consonant Mismatch Negativity task (see table above for more details). Pearson correlations were significant for 6 out of 9 electrodes in the vowel MMN paradigm (0 of 9 for consonant). A reduced amplitude of the ERP correlated with more hospitalizations, higher medication dosages and suicidal ideation.

### 3.4 Cognitive/behavioral/internet activity biomarker subgroup

Another dimension of symptoms in psychotic disorders is in the cognitive domain, with deficits, not explained by positive or negative symptoms, being present in most patients. Markers used to assess cognitive performance include output (i.e. performance) metrics in memory, attention, perception, executive function, and various other tasks. There are two studies focusing on internet search history and Facebook posting habits respectively, which are deemed as behavioral. Our search yielded 5 (12%) papers related to cognition and behavior, briefly presented in **Table 7**.

Chen et al. (50) performed two cognitive assessments, including memory, executive function and lexical tasks on 93 patients at admission and after stabilization. Using a multiple logistic regression model, the relative risk for the occurrence of relapse based on various cognitive factors was estimated. Only the preservative error in the Wisconsin Card Sorting task yielded a significant odds ratio of 2,46. Rund et al. (51) implemented a prospective design, with yearly cognitive evaluations in 207 patients (111 remained after the first year), on five basic cognitive indices, namely Working Memory (WM), Executive Function (EF), Verbal Learning (VL), Impulsivity (Im) and Motor Speed (MS). WM and VL were found to correlate with relapses within the first year.

Tao et al. (54) conducted monthly cognitive assessments on 110 patients for one year, focusing on working memory. Of the 17 cognitive markers examined at baseline, and the 4 differences (i.e. value at baseline - value at visit x), only one, the deterioration of performance in the Letter-Number Sequence (LNS) paradigm was significantly associated with relapse risk, in a binary logistic regression model. Birnbaum et al. (52, 53), retrospectively analyzed search activity and Facebook posting habits in two separate studies, using data from 51 and 42 patients respectively. In the first (52), they assembled the entire spectrum of Facebook activity of the patients, which included messages, posts, likes, photographs, shares and comments. Words related to anger, swear or death were used significantly more frequently during pre-relapse periods. The authors used Support Vector Machine (SVM) classifiers, to label periods of data as relapse or relative health data. The best performing model achieved a specificity of 0.38 and specificity of 0.71. In the second study, they focused on internet search activity, utilizing random forests, SVMs, and gradient boosting. The best performing model (gradient boosting) had a classification accuracy of 0.65 (AUC = 0.71), which significantly differed from chance level. Most important features revolved around search length and use of words from categories such as “sexual”, “anger” or “sadness”.

### 3.5 Wearables subgroup

In this section we analyze results from an emergent area of research, which concerns the application of machine learning models on passively collected data from wearable devices such as smartwatches, but also from smartphones, to identify sudden pattern breaks constituting the signature of impending relapse. In **Table 8**, the findings of 8 (19%) such studies are illustrated.

In (55, 57, 59, 61) the CrossCheck data set is analyzed, consisting of 1-year, continuous, passively collected data via smarthones (accelerometer, GPS location, speech frequency and duration, number of calls and others) from SCZ patients. Ben-Zeev et al. (55) published data from only 5 patients for demonstrative purposes. Notably, abrupt changes in GPS activity were found in a patient, who stopped spending time in their identified primary location (likely their home). The temporal onset of this sudden behavioral shift corresponded to the first signs of psychotic symptomatology. Adler et al. (57), Zhou et al. (59) and Lamichlane et al. (61) utilized various machine learning tools, such as encoder-decoder neural networks, partition around medoids, gaussian mixture models, balanced random forests, and easy ensemble models, to classify hourly data as coming from a relapse or non-relapse period. All these approaches yielded significantly different from chance level prediction accuracies, but none of the values were absolutely very high (F2 scores were calculated to assess model performance and the maximum score was 0.23 for one of the models used in (59), which can be interpreted as yielding medium predictive power). Barnett et al. (56) followed a very similar approach, using the Beiwe app to collect 15 mobility (e.g. accelerometer or GPS location) and 16 sociability features (e.g. number of phone calls) from 15 patients for 3 months. The number of anomalies, or extremely abnormal marker values was compared between patients who relapsed and those who did not. While anomaly rate was found to be 71% higher in relapsed patients, only 3 instances of relapse were recorded. Henson et al. (58) implemented a similar data acquisition procedure, recruiting 83 patients (63 provided sufficient data) for 3-12 months. Paired anomalies (i.e. anomalies occurring simultaneously in more than one feature) were found to distinguish between the state of relapse and non-relapse with a positive predictive power of 60% and a negative predictive power of 94%. Cohen et al. (62) use a similar anomaly detection approach as in (58), collecting data from 76 patients, who used a smartphone with the mindLamp app installed for 12 months. Anomaly rate was 2.12 times higher in the months preceding relapse. Of the identified anomalies, 13 or 6.9% were found to correspond to relapse. Zlantinsi et al. (60) present preliminary results (24 patients, monitored for up to 2 years) from the ePrevention study, which aimed at using passively collected heart rate, accelerometer, gyroscope and sleep data, via the use of smartwatches, to predict relapse. Various autoencoder neural network architectures were utilized with the best performing model being a Convolutional Neural Network model, which yielded an PR AUC of 0.76 (significantly different from 0.68 for random classification).

## 4 Discussion

It has been proposed that understanding the root cause of psychosis as a state may constitute a more well-defined research objective compared to the aim for a grand unification of heterogenous diagnostic constructs, such as SCZ and BP (63). Moreover, given the burden of recurrent psychosis episodes on individual patients, any progress related to relapse prediction would not only push the frontiers of current knowledge, but would also come with immense clinical benefits, allowing for early and thus more effective intervention (64). To develop a concrete strategy for individualized relapse prediction, one must first collectively assess results from diverse fields of research.

### 4.1 Genetic biomarker subgroup

Regarding genetic biomarkers, we found that 3 (21, 22, 24) of the 4 studies used traditional regression models to correlate a compiled score with number of admissions in a cross-sectional design, while 1 (23), used a prospective design and the ROC to evaluate predictive power of semi conserved networks of co-expressed genes for the state of relapse. While at first glance results from (24) seem to contradict with (21), since in (21) the genetic risk profile correlates with relapse risk whereas in (24) it does not, it must be taken into consideration that in (21), although the sample size is larger, a lower threshold is set for inclusion of single nucleotide polymorphisms in the overall score, which could lead to interpretability or overfitting issues. In (22) authors also report significant telomere shortening in relapsed individuals, however, the implication of telomeres in a wide variety of diseases may pose specificity concerns. Overall, it could be stated that although there is promise in genetic research related to relapse, it is not yet feasible to concretely predict disease trajectory based on genetic markers.

### 4.2 Blood-based biomarker subgroup

We now turn to the most thoroughly studied category of biomarkers, the diverse set of blood-based biomarkers, encompassing everything from inflammatory cytokines or hormones regulating metabolism, to nerve growth factors. Interestingly, BDNF and NGF growth factors, hypothesized to play a key role in SCZ pathogenesis (65), were conclusively ruled out as predictors of relapse, in three separate prospective studies (30, 34, 39), providing possibly the most concrete, albeit negative result covered in the present systematic review. In (25, 28, 29) the levels of various phospholipids, kynurenines and the Total Antioxidant Capacity are compared between relapsed and FEP patients at one point in time. All three studies yielded the same qualitative results. While the examined biomarkers differed significantly between patients and healthy controls, they did not differ between the relapse and FEP patient groups. Although the variation of these biomarkers in time was not assessed and thus their implication in relapse cannot be ruled out conclusively, evidence points to them being potential trait, but not state markers for relapse. In studies focusing on cytokines (27, 33, 37), only the positive correlation of Interleukin-6 with relapse is replicated (33, 37). In (37) Interferon-γ pre and post relapse values within the group of patients who relapsed were found to differ significantly. Comparisons and results from (37) are representative of the complexity of the studied phenomenon. While direct comparisons between the relapse and non-relapse groups showcased no differences in group means, longitudinal variation of IL-6 and INF-γ was found to correlate with the temporal onset of psychotic episodes. It is noteworthy that baseline values were not predictive of relapse, for neither of the two cytokines. Nevertheless, IL-6 especially should be considered as a valid candidate state marker, while it should be highlighted that it is also the most widely accepted blood-based trait marker for psychotic disorders (66, 67). Significant results are also reported in (32, 35, 36), examining white blood cells (and various ratios among subtypes), Unconjugated Bilirubin, and Vitamin D (and PTH) respectively. In the first two studies, differences in time were observed between relapse and remission, whereas in (36), relapsed patients were compared to FEP. Even though (32, 35) both report significant results, potential study limitations should not be overlooked. Firstly, specificity issues arise in both studies, since white blood cell, but also bilirubin levels may be abnormal in a wide variety of transient or chronic syndromes not related to psychiatry. Moreover, concerns related to absolute biomarker values should be considered. In (35) for instance, mean UCB levels at both relapse and remission (0.38 +/- 0.19 and 0.34 +/- 0.16), although different to each other, lie well within the normal range of 0.2 – 0.8 mg/Dl (68). Lastly (26, 31, 38), measure multiple markers, which form panels, consisting of numerous individual substances (26), a single combined index [Allostatic Load, (31)], or weighted networks (38). All three papers report significant results while the most important predictors of relapse were phenylalanylphenylalanine, systemic blood pressure, leptin, proinsulin, b-cellulin and transforming growth factor-α. However, the effects of another crucial confounding factor, which is antipsychotic treatment, should be considered, as is evident in (26). While their model consisting of 12 proteins and molecules predicted the relative time to relapse with 94.5% accuracy, a model using just BMI as a predictor achieved an accuracy of 83.4%. This could be explained by the fact that included proteins such as leptin or insulin relate to metabolism, which is affected by antipsychotic treatment. Treatment adherence has already been isolated as the single most significant clinical predictor of relapse. Therefore, it would not be irrational to hypothesize that individuals showing good compliance with medication gain more weight as a medication side effect, and experience significantly less relapses. But perhaps the most substantial limitation of the blood-based relapse prediction approach pertains to the estimation of the temporal onset of psychotic relapse. It is massively impractical to monitor blood-based markers at a sufficient frequency, to capture the onset of a phenomenon, which is acute, yet may happen at any point in time over an extremely long period. To conclude, even though there are some promising results that warrant further investigation, it cannot yet be stated that relapse prediction is possible by blood examination.

### 4.3 Neuroimaging/neurophysiological biomarker subgroup

With respect to neuroimaging, the most prominent study design revolves around predicting long term clinical outcome based on data from a baseline, structural MRI scan (40, 41, 43, 46, 47). In (40, 41), *post-hoc* comparisons between good and bad outcome patients yielded significant differences between the two groups in ventricular volume (higher in bad outcomes) and grey matter volume in the right hippocampus (lower in bad outcomes). However, studies utilizing classification models, namely SVM [in (43)] and Cox regression [in (46)] failed to achieve above chance level predictive accuracy. In (47), the Local Gyrification Index (measure of cortical folding, characteristically higher in neurodevelopmental anomalies) was significantly higher in relapsed patients, at specific loci, including the left precuneus and cuneus cortex, the isthmus cingulate gyrus, the pericalcarine cortex, and the lingual gyrus. Of the remaining 5 studies, 3 included functional MRI scans, 2 (48, 49) of which were resting state, whereas in one (42), the scans were obtained during the Semantic association retrieval task (SORT). In (48, 49) measures related to functional connectivity across multiple regions, in particular the Striatal functional connectivity index and the Global functional connectivity strength, were found to be significantly different (SCI was lower, while GFC was higher) in the group of patients experiencing psychotic relapse, hinting at impaired connectivity as a possible generative mechanism for relapse. In (42), only the behavioral component of the task, specifically reaction times, and not the imaging data, yielded significant results for relapsed versus FEP patients (relapsed patients responded slower). In a prospective PET scan study (44), temporal changes in striatal dopamine levels were found to correlate with psychotic relapse, potentially implicating dopamine autoregulation, as another contributing factor in relapse. Finally, in the only electroencephalography (EEG) study, it was shown that the amplitude of an event related potential called the phonetic Mismatch Negativity (induced by vowel change) (45), was positively correlated with re-hospitalization frequency and medication dose increase (the amplitude was significantly higher in 6 of the 9 studied electrodes in the vowel change case, but in 0 of 9 electrodes in the consonant change case. To sum up, significant group differences seem to implicate structural anomalies (e.g. ventricular volume growth), functional connectivity dysregulation (lower SCI, higher GFC), aberrant dopamine autoregulation, as well as automatic speech processing dysfunction as potential factors in relapse pathogenesis. However, it should be highlighted that the only two studies attempting *a priori* classification both failed to achieve accurate predictions. Furthermore, the same issues and confounders described for blood-based biomarkers are present in neuroimaging studies. Even though signals originating from neuroimaging scans are information-rich, it is impossible to obtain them at a frequency necessary to capture relapse onset, and thus no estimate for the temporal onset can even be formulated. To conclude, no tangible clinical benefit for relapse prediction can be claimed from neuroimaging/neurophysiological biomarker monitoring.

### 4.4 Cognitive/behavioral/internet activity biomarker subgroup

In the cognitive/behavioral biomarker section, 3 studies (50, 51, 54) pertaining to typical cognitive assessments were analyzed. Generally, they consisted of various tasks evaluating five main pillars of cognitive function, defined as Working Memory (WM), Executive Function (EF), Verbal Learning (VL), Impulsivity (Im) and Motor Speed (MS) (51). In two of these studies (51, 54) working memory deficits were the only significant predictor of relapse, while in the third (50) it was executive function hindrance, captured with the preservative error rate in the Wisconsin cart sorting task. Inconsistent results could be attributed to confounders, mainly heterogeneity in clinical manifestations, or different treatment regimens and adherence. All three studies yielded one statistically significant predictor among almost 20, which does not support detailed cognitive assessments as viable tools for efficient relapse prediction. In (52, 53) authors used data from Facebook (i.e. messages, posts, comments) and internet search activity, known to exhibit distinct patterns and tried to detect pattern breaks related to relapse. The authors report highly significant differences in the frequency of use for significant words related to “anger”, “death” or “sadness”. They also utilized supervised machine learning techniques, such as SVMs or gradient boosting, to classify a random time series as coming from a period of relapse or remission. These analyses yielded predictions that significantly differed from chance level but were not highly accurate. For reference, AUC values range from 0.5 to 1, with 0.5 representing chance level accuracy and 1 indicating complete certainty. Anything over 0.8, which was not reached, is generally considered as accurate, whereas values lying in the 0.7-0.8 range (0.71 was the value obtained from the best performing model), are considered adequate depending on context.

### 4.5 Wearables biomarker subgroup

Regarding the emerging field of relapse prediction via analysis of passively collected smartphone and smartwatch data, our search identified 8 studies conducted in the past 7 years. In (55, 57, 59, 61) various tools are used to analyze the data from the CrossCheck data set. Smartphones with the CrossCheck app installed were provided to all subjects and were utilized to gather a variety of mobility and sociability features, for instance GPS location, accelerometer data, or phone call duration and number of texts. After showcasing the potential of this approach in (55), where alterations in single features for individual subjects, for example the complete cessation of gross movements, captured with GPS sensors, seemed to closely precede relapse episodes, it was then attempted to systematically predict relapse via machine learning models trained on aggregated data (57, 59, 61). The trend observed in behavioral studies (52, 53) involving *a priori* classification via machine learning was also observed in these studies. Although results differed from chance level, the absolute predictive accuracy did not reach higher values. This tendency could be explained by heterogeneity in clinical manifestations. Using the above example with GPS sensing, it is conceivable that GPS activity would change in various ways, besides the mentioned trend of movement cessation. The exact opposite, which would be wandering, or spending little to no time in the identified primary location, is possible, while no change occurring at all is also a likely possibility. This lack of a unified direction in potential pattern breaks could mislead models and lead to misclassifications. In (56, 58, 62) the same data collection procedure is followed using the Beiwe and mindLamp applications. A similar approach, revolving around identifying anomalies, or extreme values, was used to tackle the relapse prediction problem. While the number of anomalies in the month preceding relapse was generally significantly higher, the reported positive predictive power was not very high (for instance in (62), while anomalies were 2.12 in the month preceding relapse, only 6.9% of those corresponded to a relapse episode). In (60) smartwatches were used to continuously gather heart rate, accelerometer, gyroscope and sleep data. Various neural network architectures were incorporated, with a fully connected neural network yielding the best classification results (AUC = 0.77), when data from all subjects was pooled together for model training. Overall, there seems to be promise in this emerging field of research. An important advantage we observed, is that while statistical methods and analyses may vary between studies, data collection devices and procedures, as well as extracted features, are similar, or even identical in many cases, implying that results should be generalizable. Even though till now the level of accuracy needed in clinical practice has not been achieved, these results should not be dismissed. Inclusion of more subjects and data, as well as more biometric parameters paired with model optimization, could lead to fully functional, non-invasive patient monitoring.

### 4.6 Overview of biomarkers in the current clinical framework

Undeniably, biomarker research has the potential to further our understanding of pathophysiology, as well as refine clinical practices, however, it is not clear whether biomarker testing is reliable enough to be applied already, or how it fares compared to traditional models for estimating relapse risk, which rely on clinical predictors like treatment non-adherence. To begin with, in this review we covered genetic markers, which in principle do not change in time (although in (23), networks of co-expressed genes do evolve temporally) and thus seem unfit for the study of a phenomenon such as psychotic relapse. While risk scores comprised of thousands of genes were found to correlate with adverse outcomes in one study (21), this result was not replicated in (24). Importantly, these analyses are typically performed on a population level, whereas clinical decisions are made on an individual basis. For these reasons, we do not believe genetic screening on a per-person basis is warranted. The same could be said in regard to cognitive and behavioral markers. Although cognitive deficits do become more apparent with disease progression, they do not seem to predict relapse, albeit in a small number of longitudinal studies. Only one of more than 15 cognitive markers was identified as a significant predictor of relapse in two longitudinal studies, while findings were not consistent between these studies, since the selected marker related to executive function in the first, but to working memory in the second. In the neuroimaging domain, we observed well established results in the literature, namely ventricular enlargement and low grey matter volume in certain areas for structural MRI and connectivity disruptions for functional MRI. Crucially, all significant findings originate from cross-sectional studies, where FEP patients were compared to acutely relapsed patients. However, when predictive modeling was used on baseline scan data, the accuracies did not differ from chance level. The question of whether brain atrophy is caused by or is a consequence of mental disorders is an extremely complicated one, but what we can claim is that multiple sequential scans should help to at least mark the temporal progression of the phenomenon (note that repeat scans where performed in only 1 of 8 MRI/FMRI included studies). Regarding blood-based markers, again most significant results, pertaining to vitamin D, bilirubin, and various inflammation or biochemistry markers originate from cross-sectional comparisons, while most effect sizes are relatively small. IL-6 and INF-γ were found to differ significantly within the same group during relapse and remission, however, in (37) the authors also implement predictive modeling for IL-6 values, yielding non-significant predictive accuracy. Even if there is some evidence for IL-6 as a state marker for relapse, it is less effective than current risk stratification approaches, which rely on clinical parameters. For reference, in a large retrospective cohort study, Rivelli et al. (69) report that predictors such as extrapyramidal symptoms (strongly related to treatment non-adherence, according to the authors), or substance abuse, raise the risk for relapse by 78% and 33% respectively (note that relapse incidence within 12 months was 30.52%). All things considered, we cannot stress enough that all the above fields have great potential, since any significant finding could unveil pathophysiological mechanisms, as well as ameliorate clinical intervention effectiveness. Until now, however, we do not believe that there exists strong evidence in favor of genetic, blood, or neuroimaging screening in clinical practice.

We assess studies utilizing wearable devices separately, since they put forth a radically different clinical monitoring framework. Clinical models, albeit extremely useful, can only influence clinical decision-making in certain ways. For instance, the a-priori identification of a patient as high-risk for relapse, due to lack of social support, could lead to the selection of a long-acting injectable (70), while extrapyramidal symptoms could be a reason for treatment regimen modification (71). Furthermore, risk stratification by definition does not offer a temporal estimate for the onset of an impending relapse event. In contrast, by using data from wearable devices, one can formulate predictions that are completely personalized and offer exact temporal estimates for relapse onset. Combining machine learning prediction algorithms, with modern web technologies has resulted in the development of fully streamlined patient monitoring systems, where data is analyzed on the fly and can then be visualized within a phone application, so that the patient can access analyses results. Applications such as LAMP, which are already available commercially, offer even more features, since with the consent of the patient, they can receive prompts/reminders, or share the data with their doctor and schedule an appointment if necessary. We believe the reason why these systems are not already widely used, is that the best performing models to date have only achieved sensitivities slightly over 70% with specificity in the 80-90% range. Given that these applications warn both the patient and potentially the clinician of an imminent relapse, thus eliciting drastic actions, these metrics need to be optimized further. This hurdle could be overcome with larger sample sizes and the measurement of a wider variety of markers. Of course, to incorporate wearable devices in clinical guidelines, large scale clinical trials with standardized equipment/statistical procedures will be necessary. Overall, evidence in this review points toward predictive modeling based on passively collected data as a valuable tool, to be refined and implemented in the near future. That being said, we do not deem wearables as a panacea, since changes in markers such as GPS Location could signify relapse, but offer little information regarding pathophysiology.

### 4.7 The “why” versus “when” tradeoff in psychotic relapse research

The main trade-off we observed while assimilating information from all studies arises due to the dual purpose of relapse prediction itself. On one hand, it is vital to understand pathophysiology, as in the “why” a psychosis episode occurs, and on the other hand it is independently beneficial to predict exact temporal onset, as in the “when” it will occur. Knowledge of the “when” would allow for timely intervention with existing protocols, whereas knowledge of the “why” would pave the way for novel, more effective treatment options. In studies where bioinformation-rich markers are used, (e.g. blood-based or neuroimaging), the practical difficulties entailed in data collection do not allow for monitoring at the frequency which is necessary to observe longitudinal variation. Even significant findings, mostly originating from cross-sectional comparisons, do not actually aid the formulation of predictions, but of risk assessments, which can only be applied to populations and not individuals. In studies using wearable devices, truly continuous monitoring can be achieved, and thus individualized predictive models with higher temporal resolution can be applied. However, markers such as GPS location do not offer in-depth insights into pathophysiological mechanisms. We do not consider this trade-off a logical necessity, but rather a consequence of available technologies and tools (for the record, 18 of the included studies were cross sectional, while another 10 included measurements at only two distinct time points, leaving just 14 prospective studies, 8 of which constituted the wearables group). Technological advancements, coupled with a more systematic and informed approach to tackle both the “why” and “when” questions simultaneously, are required for a concrete understanding of psychotic relapse.

### 4.8 Limitations of reviewed studies

Overall, the quality of included studies was very high, as is evident in the risk of bias assessment for each study (**Table 8**). Nevertheless, we note some limitations of the reviewed literature and the present review. To begin with, sample sizes were small, with some exceptions (21, 30)). Here, we also note the potential selection bias that could arise due to the inclusion of relapsed participants only, since some patients exhibit treatment-resistant phenotypes and are thus automatically excluded from any study involving relapse. Moreover, crucial confounding factors, such as clinical heterogeneity, treatment effects, or treatment adherence, are notoriously hard to control for, especially in longitudinal studies. A characteristic example of medication effect interference can be seen in studies where various hormones associated with metabolism, such as insulin or leptin are used independent variables. Lastly, the scope of this review did not include studies utilizing clinical predictors, thus their effectiveness, possibly in conjunction with biomarkers, was not thoroughly evaluated.

### 4.9 Conclusions and future directions

Summing up, accurate relapse prediction is a problem that yet remains to be tackled, even though remarkable strides have been achieved over the past years. The existing literature has uncovered a set of biological factors that could be implicated in the pathogenesis of relapse, as well as a novel approach for monitoring patients non-invasively and identifying the onset, or even earlier signs of relapse, via the use of data from smart devices. A combined approach, possibly incorporating even newer technological advancements, could yield more fruitful results. Crudely, the goal should be to accomplish continuous (or dense enough) monitoring, of information-rich biological signals, as non-invasively as possible and to then process the data automatically, so that clinical intervention would only be necessary when an impending relapse episode has already been identified. Other than smartphones and smartwatches, any accessory used in one’s daily routine could be modified to obtain bio signals. Headbands (72), headphones or even earpieces (73) have been altered, so that they can record brain electrical activity (small number of EEG channels). These devices are already in use in sleep research for example (74, 75)), and offer a plethora of advantages, as they are user friendly and require no technical training to be applied, the entire data collection and preprocessing procedure is streamlined in no-code GUI interfaces, and importantly they do not pose discomfort concerns for the subjects. Of course, this is only one possible example of the amalgamation of technology with biomarker monitoring. It falls upon future researchers to uncover more innovative concepts that will revolutionize our understanding of psychosis and relapse.

## Data Availability

The original contributions presented in the study are included in the article/**Supplementary Material**. Further inquiries can be directed to the corresponding author.
